# A novel NKp80-based strategy for universal identification of normal, reactive and tumor/clonal natural killer-cells in blood

**DOI:** 10.3389/fimmu.2024.1423689

**Published:** 2024-07-08

**Authors:** F. Javier Morán-Plata, Noemí Muñoz-García, María González-González, Julio Pozo, Sonia Carretero-Domínguez, Sheila Mateos, Susana Barrena, Moncef Belhassen-García, Catarina Lau, Maria Dos Anjos Teixeira, Ana Helena Santos, Ana Yeguas, Ana Balanzategui, Alejandro Martín García-Sancho, Alberto Orfao, Julia Almeida

**Affiliations:** ^1^ Translational and Clinical Research Program, Cancer Research Center (IBMCC, CSIC – University of Salamanca), and Department of Medicine, University of Salamanca, Salamanca, Spain; ^2^ Cytometry Service, NUCLEUS, University of Salamanca, Salamanca, Spain; ^3^ Institute of Biomedical Research of Salamanca (IBSAL), Salamanca, Spain; ^4^ Cell-purification Service, NUCLEUS, University of Salamanca, Salamanca, Spain; ^5^ Department of Internal Medicine, University Hospital of Salamanca, Salamanca, Spain; ^6^ Department of Infectious Diseases, University Hospital of Salamanca, Centro de Investigación de Enfermedades Tropicales de la Universidad de Salamanca (CIETUS), Salamanca, Spain; ^7^ Laboratory of Cytometry, Unit for Hematology Diagnosis, Department of Hematology, Hospital de Santo António (HSA), Centro Hospitalar Universitário do Porto (CHUP), Unidade Multidisciplinar de Investigação Biomédica, Instituto de Ciências Biomédicas Abel Salazar, Universidade do Porto (UMIB/ICBAS/UP), Porto, Portugal; ^8^ Department of Hematology, University Hospital of Salamanca, Salamanca, Spain; ^9^ Research Networking Centre Consortium of Oncology (CIBERONC), Instituto de Salud Carlos III, Madrid, Spain

**Keywords:** NKp80, NK-cells, NK-cell markers, NK-cell gating strategy, CLPD-NK/NK-LGLL, NK-cell clonality

## Abstract

**Purpose:**

Natural killer (NK) cells are traditionally identified by flow cytometry using a combination of markers (CD16/CD56/CD3), because a specific NK-cell marker is still missing. Here we investigated the utility of CD314, CD335 and NKp80, compared to CD16/CD56/CD3, for more robust identification of NK-cells in human blood, for diagnostic purposes.

**Methods:**

A total of 156 peripheral blood (PB) samples collected from healthy donors (HD) and patients with diseases frequently associated with loss/downregulation of classical NK-cell markers were immunophenotyped following EuroFlow protocols, aimed at comparing the staining profile of total blood NK-cells for CD314, CD335 and NKp80, and the performance of distinct marker combinations for their accurate identification.

**Results:**

NKp80 showed a superior performance (vs. CD314 and CD335) for the identification of NK-cells in HD blood. Besides, NKp80 improved the conventional CD16/CD56/CD3-based strategy to identify PB NK-cells in HD and reactive processes, particularly when combined with CD16 for further accurate NK-cell-subsetting. Although NKp80+CD16 improved the identification of clonal/tumor NK-cells, particularly among CD56^-^ cases (53%), aberrant downregulation of NKp80 was observed in 25% of patients, in whom CD56 was useful as a complementary NK-cell marker. As NKp80 is also expressed on T-cells, we noted increased numbers of NKp80^+^ cytotoxic T-cells at the more advanced maturation stages, mostly in adults.

**Conclusion:**

Here we propose a new robust approach for the identification of PB NK-cells, based on the combination of NKp80 plus CD16. However, in chronic lymphoproliferative disorders of NK-cells, addition of CD56 is recommended to identify clonal NK-cells, due to their frequent aberrant NKp80^-^ phenotype.

## Introduction

Natural killer (NK) cells are a cytotoxic compartment of innate lymphoid cells (ILC), which represent the first line of defense against virus-infected cells and cancer cells (1–3). In peripheral blood (PB) of healthy adults, NK-cells represent ≃ 5%-15% of all leukocytes (4). In most clinical studies, NK-cells are identified by flow cytometry (FCM) as CD56^+^ and/or CD16^+^ lymphocytes that lack CD3, to exclude T-cells. The use of this combination of markers is needed because of the absence of a single universal NK-cell marker, and the existence of two major subsets of NK-cells in normal PB: CD16^+^CD56^lo^ NK-cells (~90%) characterized by potent killing (cytotoxic) activity and CD16^-/lo^CD56^bright^ NK-cells (~5-10%) with a main immunomodulatory function (5–7). In addition, a minor population of CD16^+^CD56^-^ NK-cells (<5%) has been recently identified in healthy adults, which is significantly increased in certain chronic viral infections (e.g., HIV-1 and HCV) or during viral reactivation states (e.g., CMV) (8–13). Because of this, the use of CD56 and CD16 for the identification of the total population of human NK-cells in blood might be suboptimal, particularly in disorders associated with downregulation of CD56 or shedding of CD16 upon NK-cell activation, including reactive (12–14) and malignant (i.e., neoplastic NK-cell) (15) conditions.

CD314 (NKG2D), CD335 (NKp46) and NKp80 have all been identified as activating receptors expressed by NK-cells (16–19). The NK group 2 number D (NKG2D/CD314) receptor is a lectin-like type 2 transmembrane glycoprotein that acts as an activating and co-stimulatory receptor, leading to degranulation, cytotoxicity and cytokine production when bound to DAP10 (18, 20, 21). In turn, CD335 is an activating receptor belonging to the natural cytotoxicity receptor (NCR) family, which is expressed on the membrane of activated and non-activated NK-cells, and whose major known ligands derive from pathogens, such as the hemagglutinin protein of influenza A virus, and proteins and other ligands from other viruses (e.g., metapneumovirus, reovirus) and pathogens (e.g., *Candida glabrata* or pneumococcus) (17, 22–24). Also, ecto-calreticulin has recently been described as an endogenous, non-viral stress-ligand for CD335 (25). NKp80 is an activating receptor belonging to C-type lectin-like receptors family (16, 19, 26), also known as KLRF1, killer cell lectin-like receptor F1, which binds to the activation-induced C-type lectin (AICL) protein, and promotes NK-cell cytotoxicity against malignant T-cells, in addition to the crosstalk between NK-cells and monocytes, and interferon-γ secretion by T-cells (27–29). These (CD314, CD335 and NKp80) receptors are all expressed in virtually the entire NK-cell population (16–18), CD314 and NKp80 being also expressed on cytotoxic T-cells (21, 28, 30). Therefore, they might represent good candidate markers for the identification of the whole population of blood circulating NK-cells, even when CD56 or CD16 are downregulated in reactive conditions (i.e., in response to viral/bacterial infections) (8–13) or in clonal expansions of CD56^-/lo^ NK-cells (15).

Here, we investigated the potential utility of CD314, CD335 and NKp80 in comparison to CD56 and/or CD16, for the unequivocal and universal FCM identification of total NK-cells present in human blood from healthy controls and patients with different disease conditions, including disorders where the classical CD56 and/or CD16 NK-cell markers are lost/downregulated. Our major aim was to select the most robust NK-cell marker and marker combination to be used in routine (diagnostic) laboratories for the identification of blood circulating NK-cells.

## Materials and methods

### Patients, controls and samples

A total of 156 EDTA-anticoagulated PB samples from an identical number of subjects (91 men, 64 women; in 1 case no gender data available), with a median age of 45 years (range: 24 days to 89 years) were enrolled in this study (**Supplementary Figure S1**). For the selection of the most robust single universal NK-cell marker (i.e., with the most discriminative resolution between NK-cells and all other leukocytes), 57 PB samples from healthy donors (HD) (34 men/22 women; one case unknown) with a median (range) age of 39 years (24 days to 48 years; 6 children and 51 adults), were initially studied. Subsequently, blood samples from 80 HD (49 men/30 women; one case with missing data) with a median age of 37 years (range: 24 days–64 years; 10 children and 70 adults), including all samples evaluated in step 1, were used to select the best fluorochrome-conjugated antibody reagent against the NK-cell marker chosen in step 1 (i.e., NKp80). In a third validation step, 87 blood samples were used, which included: 43 PB samples from HD -30 adults (23 men/13 women; median age of 35 years; range: 21-48 years) and 7 children, (4 boys/3 girls; median age of 5 years; range: 24 days-14 years)- and 44 additional PB samples from adult patients with reactive processes (n=19; 13 men/6 women; median age of 61 years; range: 20-89 years), chronic lymphoproliferative disorders of NK-cells/NK large granular lymphocytic leukemia (CLPD-NK) (31, 32) confirmed to be clonal (n=17; 3 men/14 women; median age of 69 years; range: 26-86 years) and NK-cell lymphocytosis in which NK-cells showed a phenotype suspected to be clonal/pathological (**Supplementary Table S1**) but without molecular confirmation of clonality (n=8 men; median age of 57 years; range: 51-87 years), were also analyzed. A total of 3 PB samples out of the 43 HD specimens used in this step were exposed to stress conditions (prolonged periods of storage at different temperatures, as detailed below) and 3 blood samples from adult HD were *in vitro* cultured in the presence of a polyclonal stimuli, to assess the potential impact of cell-stress and acute activation on the stability of NK-cell markers, respectively. Finally, the NKp80 expression profile on T-cells and their major subsets was analyzed in 134 blood samples from HD (n=76), individuals with reactive processes (n=33) and patients with expansions of aberrant NK-cells confirmed to be clonal (n=17) or suspected to be clonal (n=8) (**Supplementary Figure S1**).

Prior to the study, all participants and/or their parents or legal representative(s), gave their written informed consent according to the Declaration of Helsinki; the study was approved (reference number CEIm-2020/12/643) by the local institutional Ethics Committee (University Hospital of Salamanca/IBSAL, Salamanca, Spain).

### FCM immunophenotypic studies

For all flow cytometry studies, fresh whole PB samples were stained following the EuroFlow standard operating procedures (SOP) for staining of cell surface membrane markers alone or in combination with intracellular antigens available at (www.euroflow.org). Immediately afterwards, stained cells were measured in either a 5-laser Cytek^®^ Aurora spectral flow cytometer (Cytek Biosciences, Fremont, CA) using the SpectroFlo^®^ software (Cytek Biosciences) or in a FACSCanto II digital flow cytometer (Becton/Dickinson Biosciences (BD), San Jose, CA) using the FACSDiva™ software (BD). EuroFlow protocols for instrument setup and data acquisition (available at www.euroflow.org) were strictly followed (33). For data analysis, the INFINICYT™ software (BD-Cytognos, Salamanca, Spain) was used.

### Selection of the most robust NK-cell marker for the identification of PB NK-cells by FCM

Based on our previous experience and the data from the literature, in a first step, three NK-cell-associated markers −CD314, CD335 and NKp80− were selected, and different monoclonal antibodies (mAb) against these markers tested (**Supplementary Table S2A**). Reagents for each of the three markers were tested in 2-4 different fluorochrome-conjugated formats (n. of samples tested): CD314 BUV615 (n=7); CD314 PE (n=5); CD335 BV421 (n=13); CD335 PE-Cy7 (n=21); NKp80 VioBright B515 (n=3); NKp80 PE (n=7); NKp80 PE-Vio615 (n=20); NKp80 PE-Vio770 (n=7). Of note, reagents against all three markers were also simultaneously tested (combined in one-single tube) in blood of 3 healthy adults, for direct comparison of their performance (**Supplementary Figure S2**). Reagent (marker/fluorochrome) performance was evaluated through their stain index (SI) values obtained (34). In all samples, the above referenced reagents were stained in combination with a panel of antibodies which additionally contained at least CD3, CD16, CD45 and CD56, for accurate identification of the target NK-cells and their corresponding appropriate negative reference cell population(s). The specific clones, fluorochrome-conjugated reagents and their source are detailed in **Supplementary Table S2A** and in **Supplementary Table S3**.

### Selection of fluorochrome-conjugated anti-NKp80 mAb clones

For the selection of the best reagent(s) (clone and fluorochrome-conjugate) against NKp80, two reagents were tested in addition to the four anti-NKp80 mAb reagents described above, for a total of six different mAb reagents. Those consisted of three different clones (REA845, 5D12 and 4A4.D10) conjugated with up to 5 different fluorochromes (Vio^®^ Bright B515, PE, PE-Vio^®^ 615, PE-Vio^®^ 770 and APC-Vio^®^ 770) (**Supplementary Table S2B**). The performance among the different anti-NKp80 reagents tested was compared based on their respective SI obtained (34).

### Validation of NKp80 as a robust universal NK-cell marker in blood of healthy donors and patients with NK-cell expansions

The utility of NKp80 for the identification of the whole population of NK-cells was assessed in blood of HD and patients, by comparing the absolute number and percentage of blood NK-cells using four different NK-cell-gating strategies (**Supplementary Figure S3**): i) conventional gating, i.e., identification of NK cells as CD3^-^ mature lymphocytes showing a typical FSC^lo^SSC^lo^CD45^hi^CD16^-/+^CD56^lo/+/++^CD3^-^ phenotypic profile for CD16 and CD56, which excluded CD16^+^CD56^-^ cells, as well as CD16^-^CD56^lo^ cells from the NK-cell gate, because of the usual contamination of these cell fractions with CD16^+^ monocytes and ILC, respectively; ii) a strategy based on NKp80 expression alone (FSC^lo^SSC^lo^CD45^hi^NKp80^+^CD3^-^) or iii) in combination with CD16-only (FSC^lo^SSC^lo^CD45^hi^CD16^-/+^NKp80^-/+^CD3^-^); and iv) a strategy based on the expression of all three CD16^+^ and/or CD56^+^ and/or NKp80^+^ markers among FSC^lo^SSC^lo^CD45^hi^CD3^-^ cells, for simultaneous identification of classical, as well as non-classical NK-cell subsets. In selected samples from patients diagnosed with reactive conditions (bacterial or viral infections), the expression patterns of the CD38, CD69 and HLADR activation-associated markers were further evaluated and related with the expression of the NK-cell-associated markers. In addition, the potential downregulation of classical CD16 and CD56 NK-cell markers together with NKp80 due to cell stress, was also evaluated in EDTA-blood samples exposed for long periods of time (+1, +4, +7 and +10 days after collection) and at different storage temperatures (room temperature and 4°C).

### *In vitro* stimulatory assays

Well-established short-term *in vitro* cell culture assays were used to directly evaluate the stability of the expression of CD16, CD56 and NKp80 on *in vitro* activated NK-cells from heparin-anticoagulated blood samples (**Supplementary Methods**) (35). In addition, to more precisely analyse the activation status after *in vitro* culturing, activation-associated markers (i.e., CD38, CD69 and HLADR) were added to the antibody panel.

### Phenotypic characterization of abnormal NK-cells in CLPD-NK and other NK-cell expansions (reactive vs suspected of being clonal)

Extended panels including (most) reagents detailed in **Supplementary Table S3** were used for a more extended characterization of the expanded and/or abnormal NK-cells found in CLPD-NK and other suspected (clonal vs reactive) NK-cell expansions, using the EuroFlow NK-cell CLPD panel and SOP (36).

### Assessment of the clonal nature of FACS-sorted NK-cell populations

Diagnosis of a chronic lymphoproliferative disorder of NK-cells/NK large granular lymphocytic leukemia (29, 30) was confirmed on highly-purified FACS-sorted NK-cells, using the polymerase chain reaction (PCR)-based HUMARA assay in women heterozygous for the androgen receptor gene, and the analysis of *STAT3* and *STAT5B* gene mutations in both women and men (37–40). In those NK-cell expansions confirmed to be clonal either by HUMARA and/or by the presence of *STAT3* or *STAT5B* somatic mutations, in the absence of NKp80 expression, T-cell lineage was ruled out by the absence of an underlying clonal TCRβ and TCRγ V(D)J gene rearrangement (41, 42).

### Statistical methods

The statistical significance of differences observed between groups for continuous variables, was assessed with the Mann–Whitney U or the Kruskal–Wallis tests, while for comparisons of proportions, the χ² test was used. Pearson’s correlation and Bland-Altman plots were employed to determine the degree of correlation and agreement between the different analytical strategies used for the identification of NK-cells, respectively. Statistical analyses and figures were carried out and plotted using the IBM-SPSS v28.0 (IBM, Armonk, NY) and GraphPad Prism V8 software packages (GraphPad Software, San Diego, CA). P-values ≤ 0.05 were considered to be associated with statistical significance.

## Results

### Selection of a universal marker for the identification of blood NK-cells by FCM

**Figure 1:**
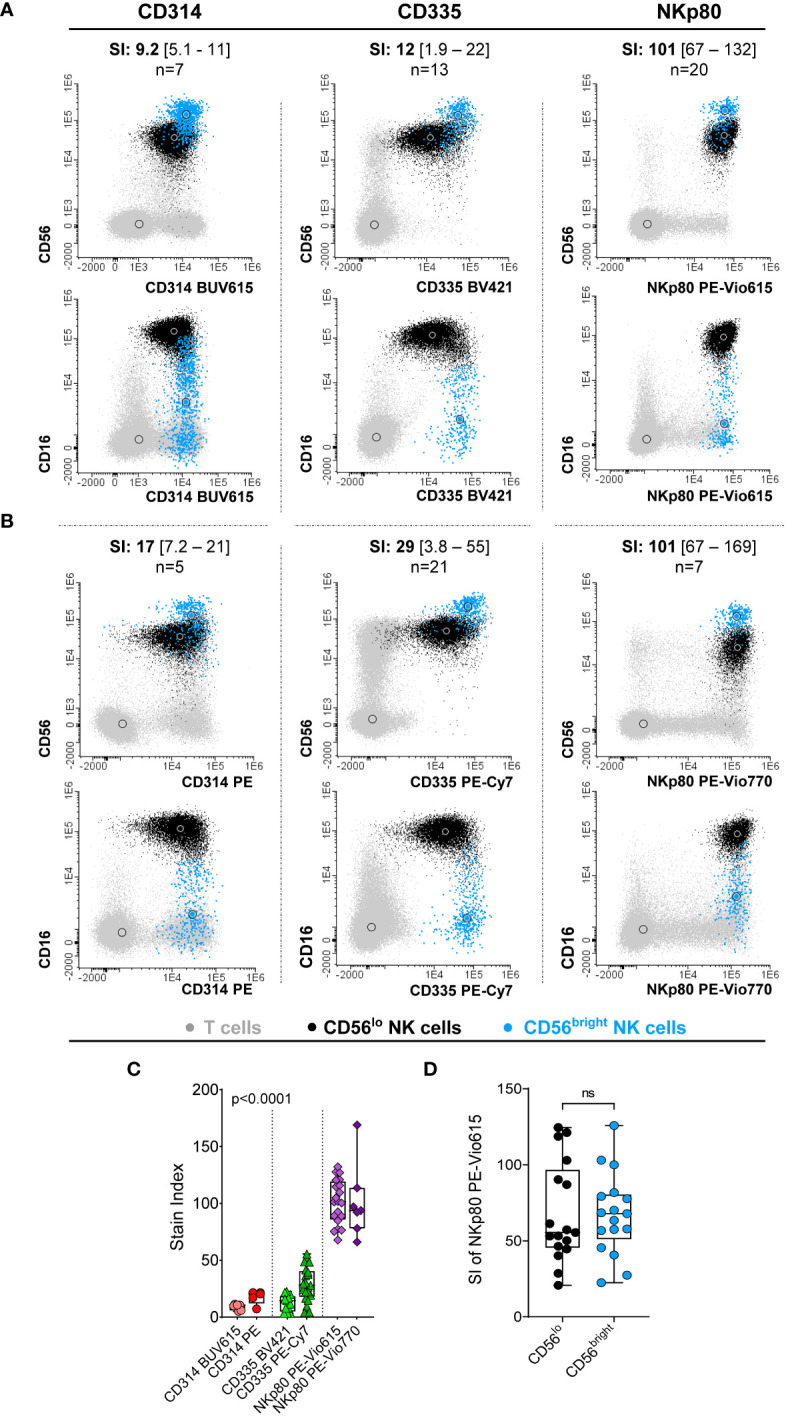
Comparative analysis of the performance of the three NK-cell candidate markers (CD314, CD335 and NKp80) tested in blood samples from healthy donors. **(A, B)** Representative images of the staining pattern observed for the CD56^lo^CD16^++^ (black dots) and CD56^bright^CD16^-/lo^ (light blue dots) NK-cell populations for the CD314 conjugates of the BUV615 (left panels in **A)** and PE (left panels in **B)** dyes, for CD335 conjugated with BV421 (middle panels in **A)** and PE-Cy7 (middle panels in **B)** and the NKp80 marker conjugated with PE-Vio615 (right panels in **A)** and PE-Vio770 (left panels in **B)**.The number of PB samples evaluated (n) and the stain index obtained for each antibody reagent tested, expressed as median [range] values, are indicated at the top of each dot plot. **(C)** Box plot showing stain index values (SI) obtained on total NK-cells for each antibody reagent displayed in panels **(A, B)** P-value (Kruskal-Wallis test) in panel **C** was mostly influenced by the SI of NKp80, where expression levels were significantly higher than those obtained for the other two markers evaluated. **(D)** Box plot representing the NKp80 PE-Vio615 stain index (SI) for the two major CD56^++^CD16^-/lo^ and CD56^lo^CD16^++^ NK-cell subsets present in blood of HD. In panels **(C, D)**, dots correspond to individual samples, while notched boxes represent the 25th and 75th percentile values; the lines inside the box correspond to median values (50th percentile) and whiskers represent minimum and maximum values. Stain indices were calculated as: MFI of the reference positive cell population – MFI of the reference negative cell population/[2 x rSD of the reference negative cell population], where total NK-cells and basophils were used as the reference positive and negative cell populations, respectively. BV, brilliant violet; BUV, brilliant ultraviolet; HD, healthy donor; MFI, median fluorescence intensity; NK, natural killer; ns, no statistically significant differences; PE, phycoerythrin; PE-Vio, phycoerythrin-Vio^R^ dye; PE-Cy7, phycoerythrin cyanine 7; rSD, robust standard deviation; SI, stain index.

Expression of CD314, CD335 and NKp80 was detected at the cell surface membrane of virtually all NK-cells from HD (98.8 ± 2.5%, 98.9 ± 1.5% and 99.9 ± 0.08%, respectively). However, CD335 was the only marker found to be specifically expressed on NK-cells, whereas CD314 and NKp80 were also expressed by populations of T-cells (**Figures 1A, B**). Despite all three markers were expressed on virtually all PB NK-cells, comparisons between the intensities of expression obtained for the three markers on (total) NK-cells revealed significantly higher SI values in every sample tested for NKp80 vs both CD314 and CD335 (p<0.001), regardless of the fluorochrome used (**Figure 1C**). These findings were further confirmed when the three markers were simultaneously assessed (**Supplementary Figure S2**). In contrast to CD314 and CD335, NKp80 on its own did not allow clear cut discrimination between the two major populations of normal CD16^-/lo^CD56^bright^ and CD16^+^CD56^lo^ NK-cells in blood, since the NKp80-associated SI values obtained for both cell subsets were highly similar (**Figure 1D**). Based on the above results, NKp80 was selected as the most robust single universal NK-cell marker in normal blood.

### Selection of anti-NKp80 mAb reagents

**Figure 2:**
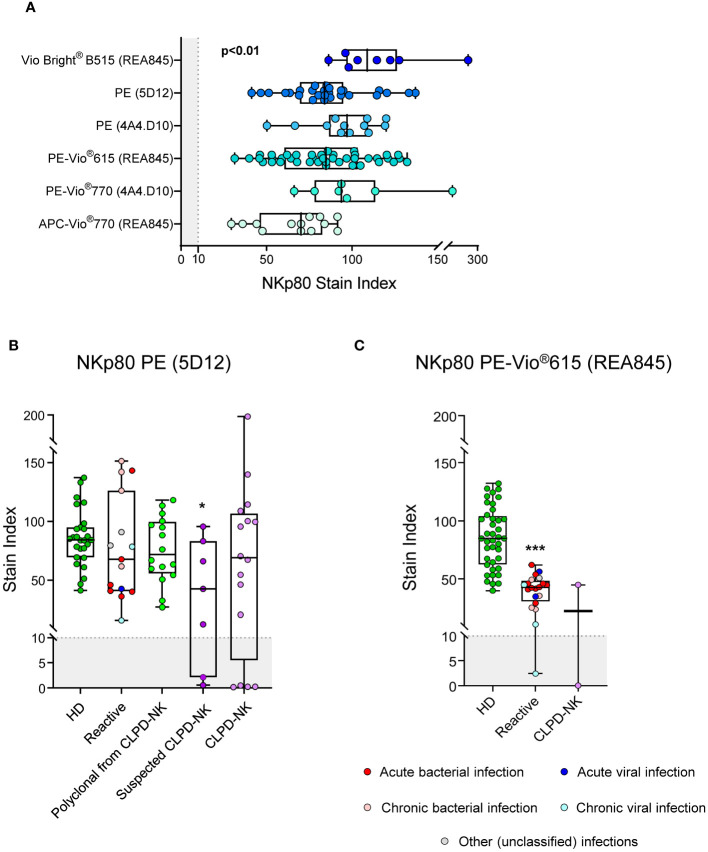
Performance of different anti-NKp80 monoclonal antibody reagents. **(A)** Box plots show stain index values obtained for the different anti-NKp80 reagent clones (REA845, 5D12 and 4A4.D10) and fluorochrome conjugates (Vio Bright^®^ B515, PE, PE-Vio^®^615, PE-Vio^®^770 and APC-Vio^®^770) tested on blood circulating NK-cells from healthy donors, identified with the conventional gating strategy (FSC^lo^SSC^lo^CD45^hi^CD16^-/+^CD56^lo/+/++^CD3^-^). The staining profiles for two NKp80 antibody reagents, conjugated with PE (clone 5D12) **(B)** and PE-Vio^®^615 (clone REA845) **(C)**, were further evaluated on blood NK-cells from patients with different pathological conditions displaying increased expanded NK-cell counts, in addition to healthy adults. Stain index values refer to total blood NK-cells. Stain indices were calculated as: MFI of the reference positive cell population – MFI of the reference negative cell population/[2 x rSD of the reference negative cell population], where total NK-cells and basophils were used as the reference positive and negative cell populations, respectively. In all box plots, dots correspond to individual samples, while notched boxes represent the 25th and 75th percentile values; lines inside the box correspond to median values (50th percentile) and whiskers represent the minimum and maximum values. In all panels, the gray shading represents the range of values at which the marker is considered negative (SI<10). In panels **(B, C)**, samples carrying reactive NK-cells are displayed in different colors, depending on the type of infection; in CLPD-NK cases, the absence of reactivity for NKp80-PE observed for the NK-cells from some of the samples, was further confirmed with at least one additional anti-NKp80 reagent (different clone and fluorochrome). P-values indicated in panel **(A)** resulted from the Kruskal-Wallis test. *p-value ≤0.05, and ***p-value ≤0.001 vs. HD. APC, allophycocyanine; CLPD-NK, chronic lymphoproliferative disorder of NK-cells; HD, healthy donor; MFI, median fluorescence intensity; NK, natural killer; PE, phycoerythrin; rSD, robust standard deviation.

Detailed comparison of six different anti-NKp80 antibody reagents for a total of three different clones and five different fluorochrome-conjugated reagents (**Supplementary Table S2B**) was subsequently performed in blood samples from 10 healthy children and 70 healthy adults. Overall, all NK-cells present in blood of HD could be identified with all six different anti-NKp80 antibody reagents tested, with SI values >70. Despite this, the performance of each individual reagent varied substantially, the VioBright B515 and PE-Vio770 conjugates (REA845 and 4A4.D10 clones, respectively) being those reagents showing the strongest and more homogeneous staining profiles (**Figure 2A**).

The pattern of expression of NKp80 on reactive and aberrant/clonal NK-cells was further investigated using two different anti-NKp80 reagents (NKp80 PE, clone 5D12 and NKp80 PE-Vio615, clone REA845). For the NKp80 PE reagent, no significant differences were observed in the expression profile of this marker on reactive/polyclonal compared to normal NK-cells. In contrast, a significant reduction of the NKp80 SI was found on NK-cells of patients with CLPD-NK and NK-cell expansions suspected to be clonal, with 2/7 (29%) and 4/16 (25%) cases, respectively, showing no NKp80 expression at all (**Figure 2B**). In contrast to the NKp80 PE (clone 5D12) reagent, when the less sensitive NKp80 PE-Vio615 reagent (clone REA845) was used, NK-cells from reactive blood samples displayed significantly lower SI levels vs normal HD cells, regardless of the underlying disease (e.g., infection). PB samples from 2 clonal NK-cell disorders were also stained with the NKp80 PE-Vio615 reagent, one of them showing an aberrant NKp80^-^ phenotype (**Figure 2C**).

### Validation of NKp80 as a robust marker for the identification of normal, reactive and clonal NK-cells

**Figure 3:**
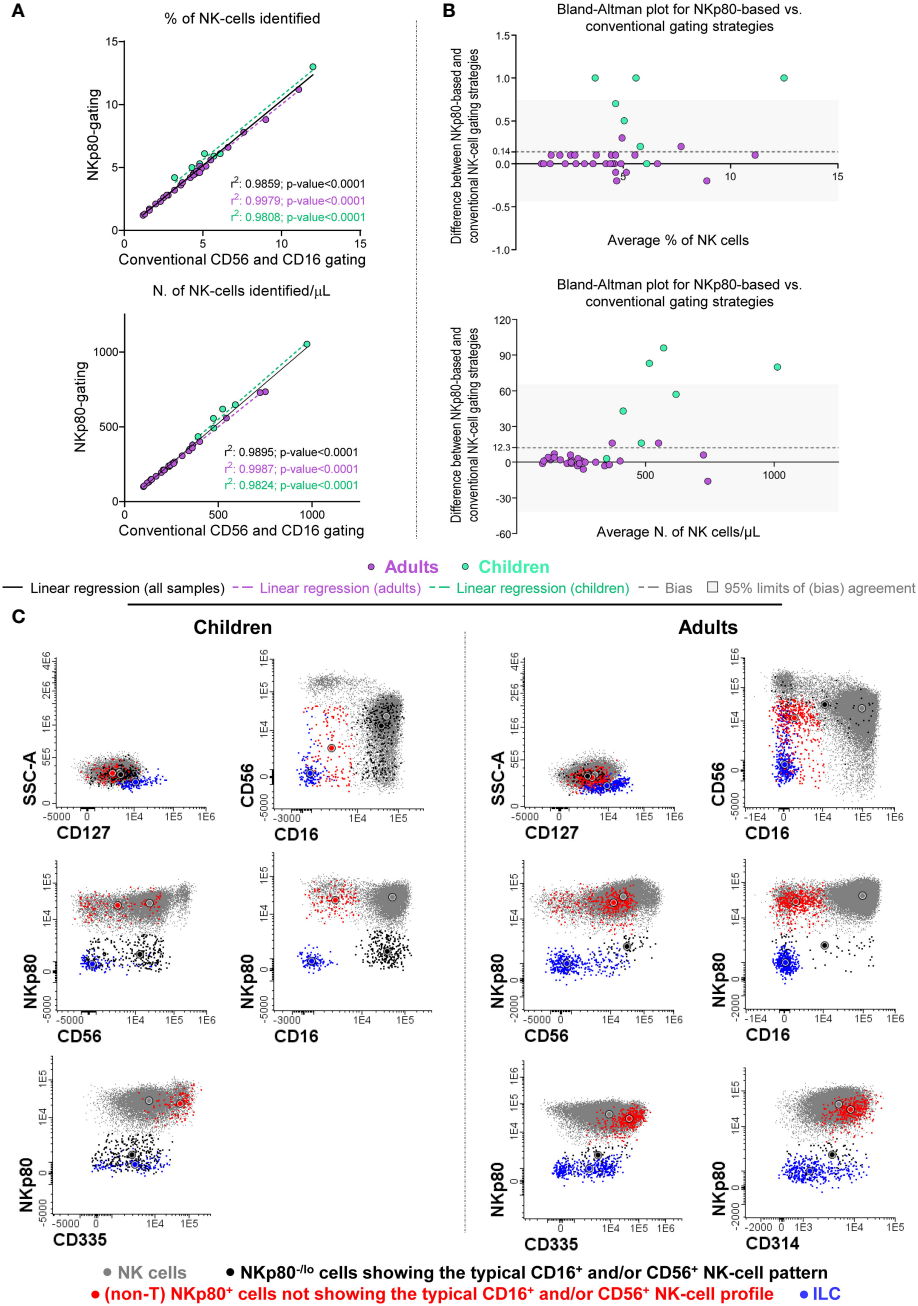
Comparison between the conventional (the CD16^+^ and/or CD56^+^) and the NKp80-based gating strategies for the identification of total NK-cells in blood samples from healthy donors. **(A)** Scatter plots for percent (from leukocytes) and absolute NK-cell numbers obtained based on the NKp80-gating strategy (y-axis) and the conventional CD16 and/or CD56 gating approach (x-axis) revealed a significantly high positive correlation between them (where r^2^ values correspond to the Pearson’s correlation coefficients) for all individual samples compared, as well as separately for adults and children. **(B)** Bland-Altman’s plots show the degree of agreement between the percentage and absolute number of NK-cells/µL identified with both the anti-NKp80-based and the conventional gating strategies. The x-axis depicts the average measurement obtained with the two data analysis strategies, while in the y-axis the differences between both methods, are shown. An y-axis value close to 0 indicates that the level of agreement is higher, y=0 represent the line of perfect (average) agreement. For the percentage on NK-cells, the mean (± SD) bias obtained was of 0.14 (± 0.31), while for the number of NK-cells/uL, the mean (± SD) bias was 12.3 (± 26.8). In Panels **(A, B)**, violet dots indicate samples collected from healthy adults and green dots those obtained from healthy children, black solid lines **(A)** indicate the linear regression of all samples studied, while violet dotted lines show the linear regression obtained for adults **(A)**, and the green dotted lines correspond to the linear regression for children **(A)**; the dashed gray lines **(B)** correspond to the Bland-Altman’s bias, and the gray band **(B)** indicates the 95% limits of a non-biased agreement (mean bias ± 1.96 SD). **(C)** Representative two-dimensional dot plots of the phenotypic profile of representative (normal) NK-cells, from healthy children and healthy adult blood, identified based on the conventional CD16^+^ and/or CD56^+^ data analysis strategy (FSC^lo^SSC^lo^CD45^hi^CD16^-/+^CD56^lo/+/++^CD3^-^) (gray dots), including NKp80^-/lo^ cells showing the typical NK-cell expression pattern for CD16 and CD56 (black dots) and other (non-T) NKp80^+^ cells that fall outside the classical NK-cell gate (red dots) for CD16 and CD56. In addition, ILC cells, identified as FSC^lo^SSC^lo^CD45^hi^CD3^-^CD19^-^CD16^-^CD56^+^CD117^het^CD127^+^, are also shown (blue dots). All cells represented were gated based on a CD45^++^CD3^-^CD19^-^ lymphoid-associated phenotype (not shown). In all plots shown in **(C)**, circles represent the MFI value obtained for each color-coded NK-cell population. ILC, innate lymphoid cells; MFI, median fluorescence intensity; N., number; NK, natural killer; SD, standard deviation.

In a first validation step, we compared the number of NK-cells (in terms of percentage from leukocytes and absolute number of cells/µL) identified in blood of healthy adults and children, using the NKp80-only gating strategy vs the conventional approach. The former strategy was based on the expression on NK-cells of NKp80, together with their typical CD45 and FSC^lo^SSC^lo^ pattern, after excluding CD3^+^ cells (**Supplementary Figure S3**). In turn, the conventional strategy was based on the characteristic pattern of expression of CD56 and CD16 in NK-cells, together with CD45, FSC/SSC and CD3 (**Supplementary Figure S3**). Our results showed that using the NKp80-only gating strategy, 4.2 ± 2.2% of leukocytes (274 ± 168 cells/µL) and 6.5 ± 2.9% of leukocytes (594 ± 227 cells/µL) corresponded to NK-cells in adults and children, respectively, compared to 4.2 ± 2.2% (273 ± 168 NK-cells/µL) and 5.9 ± 2.9% (540 ± 207 NK-cells/µL) with the conventional gating strategy, respectively. This translated into a significant correlation and an almost optimal degree of agreement between the two NK-cell gating strategies compared (**Figure 3A**). However, Bland-Altman analysis of the degree of agreement between both gating approaches revealed a mean positive bias of +0.14% and +12.3 NK-cells/µL in favor of greater NK-cell counts in blood with the NKp80-based vs the conventional gating strategies (**Figure 3B**). This bias was due to the identification of significantly higher numbers of NK-cells with the NKp80-only gating approach in children, but not in adults, because of the presence of significantly higher numbers of CD56^-^ NK-cells in most blood samples (86%) from healthy children (p<0.01 vs. adults) (**Supplementary Figure S4A**).

Of note, simultaneous staining for NKp80, CD56 and CD16 (in addition to CD3 and CD45), revealed two minor NK-cell populations which were systematically present in blood of HD: i) NKp80^-^ cells with a typical NK-cell CD16/CD56 expression profile (i.e., CD16^-/+^CD56^lo/+/++^); and ii) non-T NKp80^+^ cells outside the classical CD16^-/+^CD56^lo/+/++^ NK-cell-associated phenotype (**Figure 3C**). Both populations were more represented (p ≤ 0.05) in absolute and relative numbers in healthy children than in adults (**Supplementary Figure S4B**). A more extensive characterization of the phenotypic profiles of these two minor NK-cell populations showed that both were consistently negative for CD3, CD19 and CD127, and therefore not belonging to the T-, B- or ILC-cell compartments, respectively; further characterization of ILCs showed that they systematically lacked cytoplasmic granzyme B expression. In turn, the two minor NK-cell populations expressed variable levels of NK-cell markers, such as CD314 and CD335, particularly the NKp80^+^ CD16^-/lo^/CD56^-/lo^ cells (>97% and >94% of which were also CD314^+^ and CD335^+^, respectively) (**Figure 3C**).

**Figure 4:**
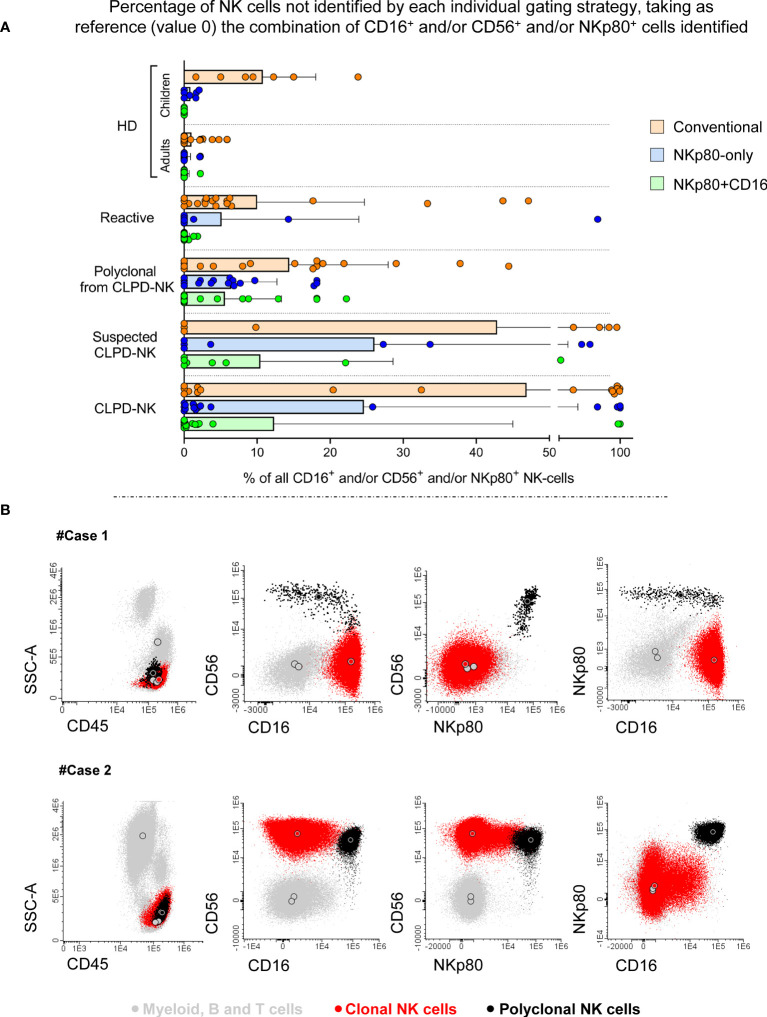
Impact of the different gating strategies used for the identification of (total) NK-cells in blood samples from both healthy donors and patients. In **(A)**, horizontal bars indicate the percentage of NK-cells that could not be identified by each of the different gating strategies (conventional, NKp80-only and NKp80+CD16), relative to the total number of NK-cells identified using all parameters simultaneously (CD16^+^ or CD56^+^ or NKp80^+^) for the identification of the minor populations of NK-cells lacking the typical CD16^+^ and/or CD56^+^ phenotypic pattern. In the horizontal bars, dots correspond to individual samples, while the size (length) of the column indicates mean values; the whiskers represent the associated (positive) SD. The following samples were used to plot the chart: 37 HD including 7 children and 30 adults, 19 reactive cases, 17 samples from CLPD-NK with residual polyclonal NK-cells cases (n=17), 8 with NK-cells suspicious of being clonal and 17 with clonal CLPD-NK cells. **(B)** Two representative CLPD-NK cases showing aberrant NKp80^-/lo^ expression profiles: #Case1 carries clonal NKp80^-^CD56^-^CD16^+^ NK-cells and #Case2 showed clonal NKp80^-/lo^CD56^+^CD16^-/lo^ NK-cells. In all flow cytometry dot plots in panel B, clonal cells are depicted in red and residual polyclonal NK-cells are shown in black. Large color-coded circles represent median values obtained for each cell population. CLPD-NK, chronic lymphoproliferative disorder of NK-cells; HD, healthy donor; lo, low; mAb, monoclonal antibody; N., number; NK, natural killer; SD, standard deviation.

In a subsequent step, we validated the incorporation of the anti-NKp80 antibody for optimal identification of total NK-cells in blood of HD and patients with different reactive or clonal NK-cell conditions, through the comparison of four different NK-cell gating strategies (**Supplementary Figure S3**). Taking into consideration that the use of all three makers, i.e., NKp80, CD16 and CD56 (following the gating strategy shown in **Supplementary Figure S3** as “CD16^+^ and/or CD56^+^ and/or NKp80^+^”) identified both classical and non-classical NK-cells (i.e., NKp80^-^ cells with a typical NK-cell CD16^+^ and/or CD56^+^ pattern and non-T NKp80^+^ cells outside the classical CD16^+^ and/or CD56^+^ NK-cell-associated profile; **Figure 3C**), we considered this strategy as the reference approach to which the other three gating strategies (**Supplementary Figure S3**) would be compared. Our aim was to ultimately select the minimum combination of markers required for an accurate identification of allblood NK-cells and NK-cell subsets. For such a comparison, we calculated the percentage of NK-cells that could not be identified with each of the other analytical strategies (conventional, NKp80-only and NKp80+CD16 gating) vs the CD16^+^ and/or CD56^+^ and/or NKp80^+^ reference approach (**Figure 4A**). For this same purpose, we further calculated the percentage and absolute number of NK-cells identified with each of the different gating strategies, compared to that obtained with the reference approach (**Supplementary Table S4** and **Supplementary Figure S5**). Overall, our results showed that, despite the added value of NKp80 was minor among healthy adults, NKp80 improved the identification of total NK-cells in both HD and the disease conditions investigated vs the conventional gating strategy. This was due to the presence of an average of 10% of NK-cells in blood of healthy children that could not be identified with the conventional gating strategy (**Figure 4A**; **Supplementary Table S4**) due to higher numbers of CD56^-^ NK-cells in children vs adults (**Supplementary Figure S4**). The additional value of using NKp80 was even more evident in reactive processes, particularly when combined with CD16 (independently of the expression of CD56), the NKp80+CD16 strategy reaching a mean percentage of NK-cells identified vs the CD16^+^ and/or CD56^+^ and/or NKp80^+^ reference approach of: 100% ± 0.51% and 99% ± 0.8% for the percentage and absolute NK-cell number vs only 90% ± 15% and 92% ± 12% with the conventional approach, respectively (**Supplementary Table S4**).

To further reinforce NKp80 as a robust marker for the identification of NK-cells, we compared its stability (i.e., downregulation) -vs that of CD56 and CD16- in blood samples from HD under stress or activating conditions. For this purpose, we exposed samples to prolonged periods of storage after collection (days +1, +4, +7, and +10) at different temperatures (4°C and room temperature, RT (22°C)), or after short-term *in vitro* stimulation. Overall, our results showed decreased SI for all three NK-cell markers over time on CD16^-/lo^CD56^bright^ and CD16^+^CD56^lo^ viable NK-cells, particularly when samples were stored at RT for several days, such decreased being more pronounced between days +1 and +4 (**Supplementary Figure S6**). Notably, the SI of CD16 on CD16^-/lo^CD56^bright^ NK-cells markedly increased at day +10, which was mainly due to a preferential death of NK-cells showing low CD16 expression levels (**Supplementary Figure S6**). Interestingly, despite the expression levels of CD16, CD56, and NKp80 on NK-cells decreased upon *in vitro* stimulation, NKp80 was the individual marker showing the lesser reduction in the SI (**Supplementary Figure S7A**). Furthermore, under real conditions of NK-cell activation in patients with reactive processes related with viral or bacterial infections, we observed that CD56^-^ NK-cells showed a more activated phenotype (HLADR^+^ and CD38^lo^), while the remaining NK-cells maintained high levels of expression of both CD16 and NKp80 (**Supplementary Figure S7B**).

NKp80 also improved the identification of residual polyclonal NK-cells in CLPD-NK patients (94% ± 6.3% vs 94% ± 7.7% NK-cells identified with NKp80 alone or in combination with CD16, respectively) compared with the conventional NK-cell gating strategy (86% ± 14%) (**Supplementary Table S4**). Of note, ≥20% of clonal NK-cells from 9/17 CLPD-NK patients (53%) could not be identified based on the conventional gating strategy, as in all of them the abnormal NK-cells completely (7/9) or partially (2/9) lacked CD56, 1 of such cases showing CD56^-^ and CD16^-^ clonal NK-cells (**Supplementary Figure S5**). This translated into poorer identification of NK-cells with the conventional approach: 53% ± 46% for the relative and 48% ± 47% for the absolute NK-cell counts vs the reference strategy (**Supplementary Table S4**). Because of this, addition of NKp80 significantly (p<0.01) improved the identification of pathological NK-cells in these disorders compared to the conventional gating strategy, particularly when combined with CD16; thus, the reference gating strategy allowed the identification of 88% ± 33% (percentage values) and 81% ± 40% (absolute cell counts) of the pathological NK-cells (**Supplementary Table S4**). However, it should be noted that in 5/17 CLPD-NK cases (29%) aberrant downregulation (low/negative) of NKp80 expression was also observed in clonal NK-cells, which prevented the identification of all NK-cells in these cases with the gating strategy based on NKp80-only (**Supplementary Figure S5**). However, once combined with CD16, the NKp80+CD16 gating strategy allowed (in NKP80^-/lo^ cases) the identification of clonal NK-cells in three CD16^+^CD56^-/+^NKp80^-^ CLPD-NK cases (illustrated for the CD16^+^CD56^-^NKp80^-^ #Case1 in **Figure 4B**), but still failed to identify clonal NK-cells in the two remaining NKp80^-/lo^ cases (2/17; 12% of all CLPD-NK) (**Supplementary Figure S5**) who had aberrant CD16^-^CD56^++^NKp80^-^ NK-cells (illustrated for #Case2 in **Figure 4B**). Consequently, only when using the gating strategy based on all three markers, all clonal NK-cells could be identified in 100% (17/17) of CLPD-NK cases (**Supplementary Figure S5**). Finally, in cases carrying aberrant NK-cells highly suspected of being clonal, similar results to those described for CLPD-NK with the conventional gating strategy were observed (**Figure 4A** and **Supplementary Table S4**). Thus, NK-cells could not be appropriately identified with the conventional approach in 4/8 cases (50%) having >50% aberrant CD16^-^/CD56^-^ NK-cells, whereas NKp80 improved their identification. However, once NKp80 plus CD16 (without CD56) were used, there were still 2/8 suspected CLPD-NK cases (25%) in which ≥20% of the suspected NK-cells could not be identified (**Supplementary Figure S5**).

### Expression of NKp80 on T-cells and their subsets

Since NKp80 is also expressed on T-cells, we further investigated the distribution of NKp80^+^ cells (in relative and absolute numbers) within total T-cells and their major blood subsets from healthy children and adults blood, as well as in patients with reactive processes and with abnormal expansions of NK-cells (**Supplementary Figure S8**). Overall, higher numbers of normal NKp80^+^ cells were found among the cytotoxic (i.e., TCD8^+^ and TCRγδ^+^) T-cells compared to TCD4^+^ cells (p<0.001), independently of age. Despite this general behavior, children showed lower numbers of NKp80^+^ T-cells within all T-cell subsets compared to adults, differences in absolute counts reaching statistical significance for total T-cells, and the major TCRαβ^+^ CD4^+^ and TCRαβ^+^ CD8^+^ T-cell subsets (**Supplementary Figure S8A**). Subsequently, we investigated the distribution of NKp80^+^ cells within the different maturational compartments of TCRαβ^+^ CD4^+^, TCRαβ^+^ CD8^+^ and TCRγδ^+^ T-cells, separately in adults and children (**Supplementary Figure S8B**). As might be expected, the percentage of NKp80-expressing cells within each maturation stage slightly increased from naïve to memory and effector memory T-cells, although differences in absolute counts did not reach statistical significance. Conversely, significantly higher relative and absolute numbers of NKp80^+^ T-cells were observed in adults vs children for virtually all maturation stages within the distinct T-cell populations (**Supplementary Figure S8B**). Finally, we compared the blood counts of normal total T, TCD4^+^, TCD8^+^ and TCRγδ^+^ cells expressing NKp80 in healthy adults and in patients with reactive disease conditions, CLPD-NK and NK-cell expansions highly suspected to be clonal. Overall, T-cells from cases with reactive NK-cells, but not from individuals with clonal or highly suspected of being clonal NK-cell expansions, showed significantly reduced numbers of NKp80^+^ cells/µL within all major compartments of T-cells analyzed (**Supplementary Figure S8C**, **Supplementary Table S5**).

## Discussion

At present, the identification of normal, reactive and even clonal NK-cells in the clinical setting relies on the combined expression of CD16 and/or CD56 on lymphocytes that do not express CD3, due to the lack of a universal NK-cell specific marker (5–7). However, previous studies have recurrently shown that this results in suboptimal identification of NK-cells, due to the lack of one or both markers in a variable percentage of all normal, reactive and clonal NK-cells (9, 12, 13, 15, 37). Here we evaluated the potential combination of new NK-cell associated markers and marker combinations based on NKp80 alone or together with CD16, and also CD56, for improved identification of the entire population of NK-cells in blood of healthy subjects and patients with different disease conditions in routine diagnostic laboratories. For this purpose, we first selected and compared the CD314, CD335 and NKp80 NK-cell-associated markers that had been reported in the literature to be expressed by virtually all human blood NK-cells in baseline (e.g., normal) conditions (16–18). Of note, from these three markers, CD314 and CD335 have been recently introduced into large spectral flow-cytometry panels as NK-cell markers (43–48), in contrast to NKp80, despite the well-known high expression levels of this later receptor on NK-cells (49). Here we show that, despite the vast majority of normal NK-cells tested positive for all three markers, NKp80 exhibited a significantly superior performance compared to the other two, evidenced by its ability to better identify NK-cells and discriminate them from other blood leukocytes. Accordingly, NKp80 showed higher and more homogeneous expression levels for all fluorochrome-conjugate reagents tested, even including those associated with the lowest SI levels (i.e., PE-Vio 615 and APC-Vio 770). Despite all the above, and in contrast with CD335, the expression of NKp80 was not specific of NK-cells, as it was also positive in a subset of T-cells. Therefore, identification of NK-cells based on NKp80 also requires the addition of CD3 to exclude T-cells, as currently done with the conventional CD16^+^ and/or CD56^+^ NK-cell gating strategy.

Once NKp80 -used together with CD3 to exclude T-cells-, was confirmed as the most robust individual marker from the three evaluated to identify NK-cells in normal blood, we subsequently compared the NKp80-based gating strategy against the conventional CD16^+^ and/or CD56^+^ approach, as the current gold standard. Despite a strong correlation and a high degree of agreement was observed between the two NK-cell gating strategies, a bias was noted in healthy children, but not in adults, due to systematically higher count of NK-cells with the NKp80-based vs the conventional approach. These differences were due to the greater number of CD56^-^ NK-cells in blood of healthy children vs adults. These results, together with our functional data on the expression of NKp80 on stressed and *in vitro* stimulated cells, confirm and extend on previous observations by Orrantia et al. on activated/reactive CD56^-^ NK-cells (9, 49). Accordingly, NKp80 was found to be a more sensitive marker than the combination of CD56 and CD16 to identify normal NK-cells, particularly in children, even under stressed conditions. Additionally, NKp80 would also be useful to exclude NK-cells from the analysis, as shown for minimal/measurable residual disease (MRD) monitoring in T-cell acute lymphoblastic leukemia (T-ALL), once combined with both cytoplasmic and surface CD3 and CD16, since NKp80 expression was found to be consistently negative in blast cells in all MRD^+^ T-ALL cases studied (n=77; unpublished results).

Despite its greater sensitivity, NKp80 on its own did not allow the discrimination between the two major populations of CD56^bright^ and CD56^lo^ NK-cells. For this purpose, simultaneous staining for NKp80, CD56 and/or CD16, in addition to CD3 and CD45, would be required. Interestingly, when we stained blood samples simultaneously for the above three NK-cell-associated markers, two minor populations of non-classical NK-cells emerged, in addition to the conventional NK-cell subsets. One population was characterized by the absence of NKp80 and a typical CD56^+^ and/or CD16^+^ pattern, while the other comprised NKp80^+^ non-T cells showing a unique CD16^-/lo^CD56^-/lo^ phenotypic profile. Today, little is known about these two minor cell populations, which showed features different from those of ILC, consistent with their NK-cell nature, as they both expressed other NK-associated markers, including CD314 and CD335. Although the former NKp80^-^ subset had not been reported so far in HD, it has been described that following *in vitro* stimulation with PMA or a cocktail of cytokines (IL-2+IL-12+IL-18), NK-cells might downregulate NKp80 expression, as also confirmed here after short-term *in vitro* stimulation, suggesting that downregulation of NKp80 might induce a negative regulatory circuit loop that would facilitate autonomous control of NK-cell responses during inflammation and infection (26). Whether or not the NKp80^-^ NK-cell subset here identified consists of an activated NK-cell population that has downregulated NKp80 expression, as well as its precise functional role, deserve further investigations. In turn, the other minor CD16^-/lo^CD56^-/lo^ subset might correspond to “non-conventional CD16^-^CD56^lo^ NK-cells” previously reported in the literature, whose functional role remains unknown (6, 7, 11). Interestingly, our data demonstrated that both subsets of non-classical blood NK-cells are more abundant in children vs adults, which might contribute to unravel their functions.

In addition to the presence of a minor physiologic population of NKp80^-^ NK-cells, we also showed here for the first time that clonal, but not reactive, NK-cells frequently lack NKp80 expression. These findings highlight the notion that NKp80 is not a universal marker for NK-cells, further emphasizing the need for new gating strategies to identify the whole population of normal, reactive and clonal NK-cells. For this purpose, we compared four different analytical strategies, considering as the reference approach that based on simultaneous assessment of NKp80, CD56 and CD16 for the identification of NK-cells, that allows identifying the classical and non-classical NK-cell subsets. Globally, our results revealed that in HD, particularly in children, incorporation of NKp80 into FCM data analysis enhances the identification of the total NK-cell population, as compared to the conventional CD16^+^ and/or CD56^+^ strategy. In fact, the optimal strategy associated with the lowest number of NK-cell markers was the combination of NKp80 plus CD16. Likewise, this latter approach also performed optimally in subjects with reactive processes, where the presence of higher numbers of CD56^-^ NK-cells compared to healthy adults has been extensively documented (12, 13, 50–52). However, in reactive processes we observed lower NKp80 levels, in addition to lower positivity for CD56, in reactive vs normal blood NK-cells. This finding might be due to downregulation of NKp80 expression during NK-cell activation (53), as also demonstrated here via short-term *in vitro* stimulation assays. This reinforces the need of CD16 in addition to NKp80, for a more accurate identification of normal and reactive NK-cells. Finally, the performance of the different analytical strategies was compared in patients with both CLPD-NK and NK-cell lymphocytosis highly suspected of being clonal, in which loss of CD16 and CD56 has been recurrently reported as a frequently tumor-associated aberrant phenotype (14, 15, 54–57). Overall, our results revealed that none of the single-marker strategies or double NK-cell marker gating approaches could identify the whole population of clonal NK-cells in every individual patient, except for the reference approach (i.e. CD16 *plus* CD56 *plus* NKp80). Thus, the inclusion of NKp80 greatly enhanced the identification of clonal and potentially clonal NK-cells vs the conventional strategy. However, aberrant NKp80^-^ clonal (and suspected of being clonal) NK-cells were also found in a significant fraction of the cases. Therefore, simultaneous inclusion of all three CD56, CD16 and NKp80 markers is recommended in the NK-cell gating panel to identify all aberrant/clonal NK-cells, because aberrant downregulation of CD16 and NKp80, and not just CD56, is also frequently observed in CLPD-NK cases.

To our knowledge, this is the first report in which aberrant downregulation of NKp80 expression (i.e., negativity) is described in between one fourth and one third of CLPD-NK cases, pointing out its potential utility as a surrogate marker of NK-cell clonality, as downregulation of this marker was not found in normal and reactive NK-cells. Due to the current limitations in assessing NK-cell clonality because of: i) the restricted value of the HUMARA technique (solely applicable to women that are heterozygous for this androgen receptor gene) (39, 40); ii) the low prevalence of *STAT3/5b* mutations, as well as the recently described *TET2* mutations (restricted to 33%-38% and 28%-34% of CLPD-NK cases, respectively) (38, 58, 59); and iii) the uneven worldwide availability of these techniques across diagnostic laboratories, then the aberrant downregulation of NKp80 here reported in CLPD-NK might be of great value as a new reliable indicator of NK-cell clonality. This aberrancy would be particularly useful for diagnosis, considering that the suspected cells commonly express other NK-cell related markers that show similar expression profiles in clonal, reactive and normal NK-cell, such as cytoplasmic granzyme B (37). Altogether, these data indicate that, despite combined assessment of CD16 plus NKp80 would be enough for the identification of all NK-cells and their subsets in blood of HD and patients with reactive conditions, CD56 should also be added in cases suspected of NK-cell clonality for a more accurate identification of clonal (aberrant) NK-cells.

Similarly to the NKG2D receptor (60–62), NKp80 expression has been shown not to be restricted to NK-cells, but expressed also by some subsets of CD56^+^, CD8^+^ and TCRγδ^+^ T-cells (16, 28, 63, 64). Here we confirm and extend on these observations through the demonstration of the existence of NKp80^+^ cells at different maturation stages among both TCD8^+^ and TCRγδ^+^ T-cells, as well as CD4^+^ T-cells from children and adults. As expected for a cytotoxicity-associated marker (16, 28, 29, 64), NKp80 showed increased expression among normal blood T-cells from adults vs children, with progressively higher levels along the different maturation stages of normal blood T-cells, similarly to the pattern observed for other cytotoxicity markers, like granzyme B, perforin or CD57 (65–69).

In summary, here we described and validated a new strategy for the identification of normal and reactive NK-cells, based on the combination of CD16 and NKp80 (after excluding CD3^+^ T-cells) which improves the conventional CD16^+^ and/or CD56^+^ NK-cell gating strategy currently used by most diagnostic laboratories. However, in cases with abnormally expanded, e.g., clonal, NK-cells, addition of CD56 to the NKp80+CD16 combination should be considered for more accurate identification of NK-cells and the discrimination between normal and clonal NK-cell compartments, due to aberrant expression (i.e., absence) of NKp80 by clonal NK-cells in a significant proportion of CLPD-NK patients.

## Data availability statement

The raw data supporting the conclusions of this article will be made available by the authors, without undue reservation.

## Ethics statement

The studies involving humans were approved by Ethics Committee of the University Hospital of Salamanca/IBSAL, Salamanca, Spain. The studies were conducted in accordance with the local legislation and institutional requirements. Written informed consent for participation in this study was provided by the participants’ legal guardians/next of kin.

## Author contributions

FM: Conceptualization, Data curation, Formal analysis, Investigation, Methodology, Resources, Software, Supervision, Validation, Visualization, Writing – original draft, Writing – review & editing. NM: Data curation, Formal analysis, Methodology, Writing – review & editing. MG: Data curation, Methodology, Writing – review & editing. JP: Data curation, Formal analysis, Methodology, Writing – review & editing. SC: Data curation, Formal analysis, Methodology, Writing – review & editing. SM: Data curation, Methodology, Writing – review & editing. SB: Data curation, Formal analysis, Methodology, Writing – review & editing. MB: Methodology, Resources, Writing – review & editing. CL: Methodology, Resources, Writing – review & editing. MT: Writing – review & editing, Methodology, Resources. AS: Methodology, Resources, Writing – review & editing. AY: Methodology, Resources, Writing – review & editing. AB: Data curation, Formal analysis, Methodology, Writing – review & editing. AG: Methodology, Resources, Writing – review & editing. AO: Conceptualization, Data curation, Formal analysis, Investigation, Methodology, Project administration, Resources, Software, Supervision, Validation, Visualization, Writing – original draft, Writing – review & editing, Funding acquisition. JA: Conceptualization, Data curation, Formal analysis, Funding acquisition, Investigation, Methodology, Project administration, Resources, Software, Supervision, Validation, Visualization, Writing – original draft, Writing – review & editing.

## Group member of EuroFlow consortium

F. Javier Moran-Plata, Catarina Lau, Maria Dos Anjos Teixeira, Ana Helena Santos, A Orfao, and J. Almeida are members of the EuroFlow Consortium (www.euroflow.org).
