# Supplementary material for: A novel NKp80-based strategy for universal identification of normal, reactive and tumor/clonal natural killer-cells in blood
Source: Front Immunol. 2024 Jul 8;15:1423689. doi: 10.3389/fimmu.2024.1423689 (PMC11260609; doi:10.3389/fimmu.2024.1423689)
Supplement: Supplementary file 1 [file DataSheet_1.pdf]

SUPPLEMENTARY MATERIALS

TITLE: **"A novel NKp80-based strategy for universal identification of normal, reactive and tumor/clonal natural killer (NK)-cells in blood"**

Authors: F. Javier Morán-Plata[1,2], Noemí Muñoz-García[1,2], María González-González[1,2], Julio Pozo[1,2], Sonia Carretero-Domínguez[1,2,3], Sheila Mateos[1,4], Susana Barrena[1,2], Moncef Belhassen-García[5,6], Catarina Lau[7], Maria Dos Anjos Teixeira[7], Ana Helena Santos[7], Ana Yeguas[8], Ana Balanzategui[8,9], Alejandro Martín García-Sancho[1,3,8,9], Alberto Orfao[1,2,3,9†] and Julia Almeida*[1,2,3,9†], on behalf of the EuroFlow Consortium.

METHODS

In vitro stimulatory assays. For direct assessment of the expression pattern of CD16, CD56 and NKp80 markers under cell-activation conditions, short-term *in vitro* cell culture assays were performed (1). Briefly, for each of the heparin-anticoagulated whole blood samples used, two aliquots were made. One was used as a control and the other (hereafter stimulated sample) was treated with 0.025µg/mL of phorbol 12-myristate 13 acetate (PMA) (Sigma-Aldrich Inc) plus 1µg/mL of ionomycin (Sigma-Aldrich Inc). The two aliquots were incubated for 4 hours (37ºC at 5% CO_2 and 95% humidity). Immediately afterwards, they were washed and stained using a panel with the antibodies of interest *plus* four activation-related markers (CD38, CD62L, CD69 and HLADR) (Table S3), following the EuroFlow standard operating procedure (SOP).

TABLES

Table S1. Clinical, biological and phenotypic data of PB NK-cells from patients with suspicion of chronic lymphoproliferative disorder of NK-cells.

	Case #1	*Case #2*	*Case #3*	*Case #4*	*Case #5*	*Case #6*	*Case #7*	*Case #8*
Age (y)	70	57	87	51	ND	78	56	55
Sex	M	M	M	M	M	M	M	M
WBC (x10^9 leukocytes/L)	ND	13.3	2.3	13.1	13.6	5.0	13.3	15.8
% of NK-cells	6	**45**	0.6	**55**	**40**	**42**	7	5
N. of NK-cells (x10^9/L)	ND	**5.99**	0.014	**7.18**	**5.37**	**2.10**	**0.98**	0.75
	Phenotype of NK-cells							
CD2	+	+	+	+	**-**	+	**+lo**	**++**
CD5	-	-/+lo	-	-	-	-	-	**+**
CD7	**+lo**	+	+	**-/+lo**	+	**+lo**	**+lo**	**+lo**
CD8	+(40%)	**-**	**++**	**-**	**-**	NA	**-**	**+**
CD11c	-	**+**	**+**	-	NA	**+**	**+**	-
CD16	**+lo**	+	-/+lo	+	+	**+(90%)**	+	+
CD25	-	-	-	-	NA	-	-	-
CD26	-	-	-	-	NA	-	-	-
CD56	+	**-**	++	**-/+lo**	+	**-**	**-**	+
CD57	++	**-**	-	+	NA	**-**	**-**	+
CD94	+	**+**	+	**+**	**+**	**-**	**+**	**+**
CD127	-	-	NA	NA	NA	NA	NA	NA
HLADR	-	-	+	-	NA	-	-	-
cyGranzyme B	+	+	-	+	NA	+	+	+
KIRs	+(44%)	+	**+**	NA	NA	**-**	+(17%)	+(5%)
NKp80	+	**-**	**-/+lo**	+	+	**- (30%)**	**- (15%)**	+
cyPerforin	+	+	-	+	NA	+	+	+

The percentage of NK-cells is referred to all PB leucocytes. Normal values in healthy adults for CD56^{bright} and CD56^{lo} NK-cells were 0.005 (0.001-0.025) and 0.35 (0.12-0.77), respectively, expressed as median and range (minimum-maximum values) of cells (x10^9/L) (unpublished results). As for KIR receptors, CD158a/b/e/g/h/k were studied in cases #1, #2 and #3, CD158b/e in cases #6 and #7, and CD158b was the only KIR evaluated in case #8. Expanded numbers of NK-cells, as well as those markers that were aberrantly expressed by NK-cells are highlighted in bold. Positive (+) and negative (-) signs denote presence and absence of antigen expression, respectively. Next to the positive sign (+) the percentage of NK-cells positive for that marker is shown only in case the marker was not expressed by 100% of the suspected NK-cell population. In case #3, the suspected clonal population was CD56^{bright}, and therefore a CD16$^{-/+lo}$CD57^{-}CD94^{+} profile was expected, while KIR expression is considered as an aberrant phenotype in these cells.

Abbreviations (alphabetical order): het, heterogeneous; cy, cytoplasmic; KIRs, killer-cell immunoglobulin-like receptor; lo, low expression levels; M, male; L, liter; N., number; NA, not analyzed/not available; ND, no data; NK, natural killer; PB, peripheral blood; WBC, white blood cell; y, years.

Table S2. Fluorochrome-conjugated monoclonal antibody reagents tested for (A) the selection of the most robust candidate (single) marker for the identification of total NK-cells in blood of healthy donors and (B) for the selection of the most discriminative anti-NKp80 fluorochrome-conjugated monoclonal antibody reagent.

(A)

Marker	*Clone*	*Fluorochrome*	*Source*	*Cat. Reference*
CD314	1D11	BUV615	BD	751232
	1D11	PE	Immunostep	314PE-100T
CD335	9E2/NKp46	BV421	BD	564065
	9E2	PE-Cy7	eBioscience	25-3359-42
NKp80	REA845	Vio® Bright B515	Miltenyi	130-112-777
	5D12	PE	BD	566329
	REA845	PE-Vio® 615	Miltenyi	130-112-587
	REA845	PE-Vio® 770	Miltenyi	130-105-068

(B)

Marker	*Clone*	*Fluorochrome*	*Source*	*Cat. Reference*
NKp80	REA845	Vio® Bright B515	Miltenyi	130-112-777
	5D12	PE	BD	566329
	4A4.D10	PE	Miltenyi	130-125-238
	REA845	PE-Vio® 615	Miltenyi	130-112-587
	REA845	PE-Vio® 770	Miltenyi	130-105-068
	REA845	APC-Vio® 770	Miltenyi	130-112-593

Abbreviations (alphabetical order): APC, allophycocyanine; BD, Becton/Dickinson Biosciences (San José, CA); BUV, Brilliant Ultraviolet; BV, Brilliant Violet; Cat, catalog; Cy, cyanine; NK, natural killer; PE, phycoerythrin.

Table S3. List of fluorochrome-conjugated monoclonal antibody reagents used for the identification of different T- and NK-cell subsets in all experiments performed in this study.

Marker	*Clone*	*Fluorochrome*	*Source*
CD2	TS1/8<br>RPA-2.10	PacB<br>FITC	BioLegend<br>BD
CD3	SK7	BV786; PerCP-Cy5.5	BD
cyCD3	REA613	PE-Vio770	Miltenyi
CD4	SK3	PacB<br>BV605	BioLegend<br>BD
CD5	L17F12	FITC; APC; PerCP-Cy5.5; RB780	BD
CD7	M-T701	BUV661; FITC	BD
CD8	RPA-T8<br>SK1	BUV395; BV650; PE-CF594<br>APCH7	BD
CD11c	SHCL-3	APC	BD
CD16	3G8	PacB; BV711	BD
CD19	SJ25C1<br>J3-119	BUV737; APCH7<br>PE-Cy7	BD<br>Beckman Coulter
CD25	MA-251	PE-Cy5	BD
CD27	MT271	BV421	BD
CD28	CD28.2	BV650<br>PerCP-Cy5.5	BD<br>BioLegend
CD38	HB7	APC Fire810	BioLegend
CD45	HI30	OC515<br>PerCP; AF700	BD-Cytognos<br>BD
CD45RA	HI100	BV510;	BD
CD56	NCAM16.2<br>5C9<br>N901<br>REA196	BUV737; BUV563<br>PE<br>PE-Cy7<br>APC-Vio770	BD<br>BD-Cytognos<br>Beckman Coulter<br>Miltenyi
CD57	HNK1	FITC	BD
CD62L	DREG56	BV605; BV650; SparkNIR685	BioLegend
CD69	FN50	PE Fire640	BioLegend
CD94	HP-3D9	BUV395; APC	BD
CD117	104D2	PE	BD
CD127	HIL-7R-M21	BV750; BV711	BD
CD158a/h/g	HP-MA4	eFluor 450	eBioscience
CD158b	CH-L	BUV563; BU737	BD
CD158e	REA168	FITC; APC; PE-Vio770	Miltenyi
CD158e/k	REA970	FITC; PE-Vio770	Miltenyi
CD158k	13E4	PE	InnatePharma
cyGranzyme B	GB11	PE	Sanquin
cyPerforin	δG9	FITC	BD
HLADR	L243	PacB; PE Fire810	BioLegend
TCRγδ	11F2	BUV737; PE-Cy7<br>PacB	BD<br>BD-Cytognos
Cell viability	NA	FVS780	BD

Abbreviations (alphabetical order): AF700, Alexa-Fluor 700; APC, allophycocyanine; APCH7, allophycocyanine hilite7; BD, Becton/Dickinson Biosciences (San José, CA); BUV, brilliant ultraviolet; BV, brilliant violet; cy, cytoplasmic; FITC, fluorescein isothiocyanate; FVS, fixable viability stain; NA, not applicable; NIR, near-infrared; OC, orange Cytognos;

PacB, Pacific Blue; PE, phycoerythrin; PE-CF594, phycoerythrin-cyanine-based fluorescent dye number 594; PE-Cy5, phycoerythrin-cyanine 5; PE-Cy7, phycoerythrin-cyanine 7; PerCP, peridinin chlorophyll protein; PerCP-Cy5.5, peridinin chlorophyll protein-cyanine 5.5; RB, RealBlue.

Table S4. Percentage of NK-cells identified using the different individual gating strategies, from those obtained with the reference strategy (in percent and absolute numbers) based on all NK-cell-associate markers and FCM parameters evaluated ($FSC^{lo}SSC^{lo}CD45^{hi}CD16^{-/+}CD56^{-/lo/++}NKp80^{-/+}CD3^{-}$).

% of NK-cells identified from all blood NK-cells

		HD		Reactive processes	Polyclonal from CLPD-NK	Suspected CLPD-NK	CLPD-NK
		Children	*Adults*				
% of NK-cells from all	Conventional	89±7 (76-98)	99±1.8 (94-100)	90±15 (53-100)	86±14 (56-100)	57±44 (2.7-100)	53±46 (0.29-100)
	NKp80 only	99±0.90** (98-100)	100±0.67 (98-100)	95±19 (18-100)	94±6.3 (82-100)	74±31* (25-100)	75±41** (0-100)
	NKp80 + CD16	100***	100±0.56 (98-100)	100±0.51** (98-100)	94±7.7 (78-100)	90±18**** (49-100)	88±33**** (0.05-100)
N. of NK-cells/µL	Conventional	91±4.5 (85-97)	99±1.4 (95-100)	92±12 (55-100)	83±15 (57-100)	51±44 (2.7-100)	48±47 (0.28-100)
	NKp80 only	99±0.76** (98-100)	100±0.49 (98-100)	98±3.8 (84-100)	93±6.1* (81-100)	67±38* (0.25-100)	73±44** (0-100)
	NKp80 + CD16	100±0.3** (99-100)	100±0.55 (98-100)	99±0.83* (98-100)	94±8.1* (79-100)	88±19**** (48-100)	81±40**** (0-100)

Results expressed as mean±SD (range) percentage values of NK-cells from all NK-cells identified with the reference $CD16^{+}$ and/or $CD56^{+}$ and/or $NKp80^{+}$ and $CD3^{-}$ gating approach; a value = 100 indicates that the corresponding gating strategy identifies the same number of NK-cells than the reference strategy. "Conventional" denotes the gating strategy based on the classical $FSC^{lo}SSC^{lo}CD45^{hi}CD56^{lo/+/++}CD16^{-/+}CD3^{-}$ NK-cell profile used for the identification of total NK-cells. For all other strategies used, the $FSC^{lo}SSC^{lo}CD45^{hi}CD3^{-}$ common pattern was also followed, in combination with positivity for NKp80 alone or together with CD16. *p-value ≤0.05, **p-value ≤0.01, ***p-value ≤0.001 and ****p-value ≤0.001 vs. the conventional gating strategy.

Abbreviations (alphabetical order): CLPD-NK; chronic lymphoproliferative disorder of NK-cells; HD, healthy donor; N., number; NK, natural killer; SD, standard deviation.

Table S5. Expression of NKp80 on blood circulating T-cells and their major subsets from healthy adults and patients with reactive, suspected CLPD-NK or CLPD-NK.

		HD *(n=66)*	***Reactive process*** *(n=33)*	***Suspected CLPD-NK*** *(n=8)*	***CLPD-NK*** *(n=17)*	***P-value***
% of NKp80⁺ cells*	**Total T cells**	13 (7.3-18)	9.5 (4.9-14)	17 (8.6-23)	11 (6.1-18)	NS
	**CD4⁺**	1.5 (0.41-3.4)	0.82 (0.29-2.2)	1.6 (0.45-5.5)	2.4 (1.1-6.0)	NS
	**CD8⁺**	26 (19-40)	**18 (12-33)**	32 (25-39)	30 (14-38)	**≤0.05**[a]
	**TCRγδ⁺**	49 (33-60)	41 (19-56)	NA	NA	NS
N. of NKp80⁺ cells/μL	**Total T cells**	176 (81-252)	**65 (19-175)**	260 (148-851)	104 (37-383)	**≤0.001**[a]
	**CD4⁺**	11 (23.5-26)	**2.8 (1.1-13)**	15 (3.7-91)	8.9 (8.6-59)	**≤0.01**[a]
	**CD8⁺**	112 (59-181)	**39 (16-130)**	210 (126-761)	73 (22-248)	**≤0.01**[a]
	**TCRγδ⁺**	40 (11-61)	**12 (3.0-24)**	NA	NA	**≤0.01**[a]

Results expressed as median (range) values. Statistically significant differences found (only) between NK-cells from cases with reactive processes and normal (HD) NK-cells are highlighted in bold ([a]). * Percentage of NKp80$^+$ cells within the corresponding T-cell subset.

Abbreviations (alphabetical order): CLPD-NK; chronic lymphoproliferative disorder of NK-cells; HD, healthy donor; N., number; NK, natural killer; NA, not analyzed/not available; ns, no statistically significant differences found (p-value > 0.05); PB, peripheral blood.

FIGURES

TOTAL SAMPLES (n=156)

- **HD (n=98)**
  - Children (n=10)
- **Reactive process (n=33)**
- **Suspected CLPD-NK (n=8)**
- **CLPD-NK (n=17)**

Step 1: Comparison between CD314, CD335 and NKp80 NK-cell markers (vs CD56 and /or CD16) (n=57)

- **HD (n=57)**
  - Children (n=6)
  - Adults (n=51)

Step 2: Selection of an appropriate NKp80 fluorochrome conjugate (n=80)

- **HD (n=80)**
  - Children (n=10)

Step 3: Validation of NKp80 as a universal NK-cell marker (n=87)

- **HD (n=43)**
  - Children (n=7)
  - Adults (n=36)
- **Reactive process (n=19)**
- **Suspected CLPD-NK (n=8)**
- **CLPD-NK (n=17)**

Step 4: Expression profile of NKp80 on T cells (n=134)

- **HD (n=76)**
  - Children (n=10)
- **Reactive process (n=33)**
- **Suspected CLPD-NK (n=8)**
- **CLPD-NK (n=17)**

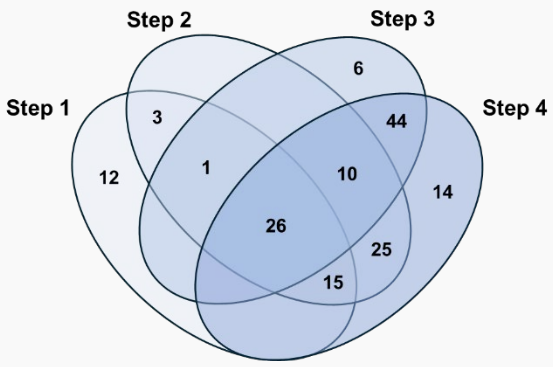

Figure S1 | Flowchart illustrating the number of PB samples (from an identical number of subjects) included in this study, and those used in each of the different experimental steps. Samples labelled as "reactive process", "suspected CLPD-NK" and "CLPD-NK" contained reactive NK-cells, aberrant NK-cells suspicious of being clonal, and clonal NK-cells, respectively, all collected from adult patients. The number of blood samples simultaneously used in the different steps is shown in the Venn diagram displayed in the lower panel.

Abbreviations (alphabetical order): CLPD-NK; chronic lymphoproliferative disorder of NK-cells; HD, healthy donor; NK, natural killer; PB, peripheral blood.

CD314	CD335	NKp80
SI = **15** [7.2 - 20]	SI = **27** [22 - 30]	SI = **104** [86 - 128]

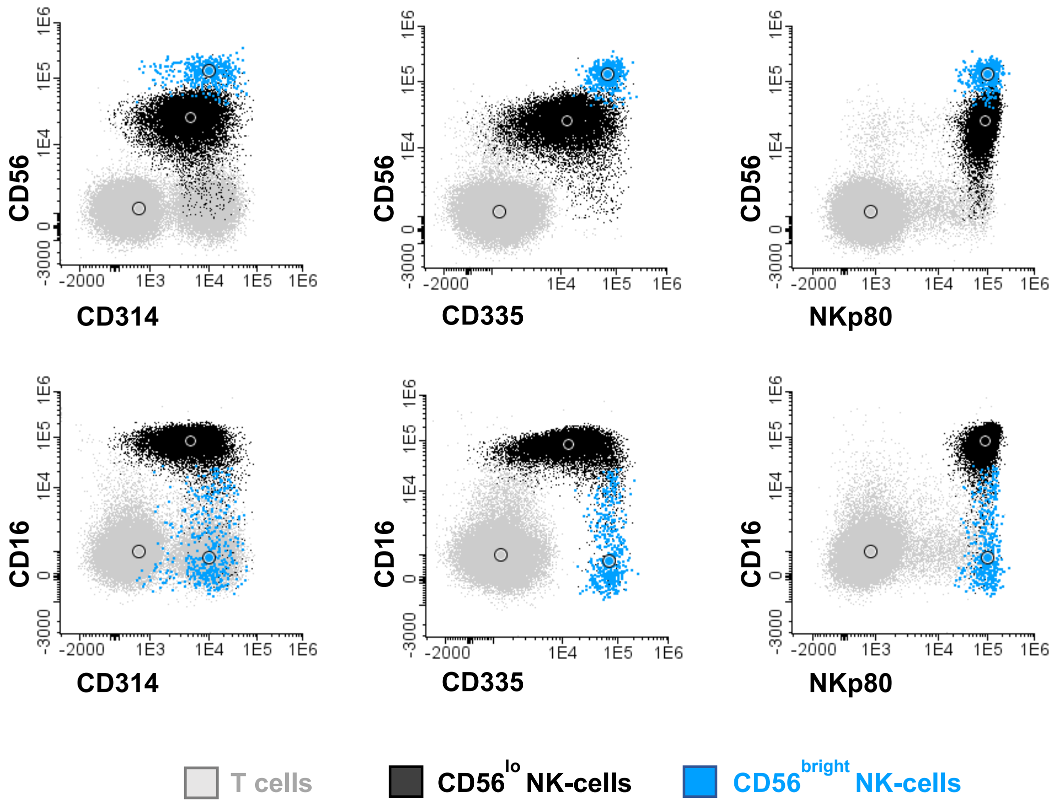

Figure S2 | Performance of the three reagents tested for the selection of a universal (robust) NK-cell marker, combined in a single-tube to directly compare their pattern and levels of expression on blood circulating NK-cells (n=3, healthy adults). Representative dot plots from one of the three blood samples are shown, where the antibodies tested against each of the markers were conjugated with bright fluorochromes (CD314 PE, CD335 PE-Cy7 and NKp80 VioBright B515). The stain index (SI) of each marker, expressed as median (range) values, is shown at the top. Stain indices were calculated using the following formula: ($MFI_{POSITIVE\ POPULATION}$ – $MFI_{NEGATIVE\ POPULATION}$) / (2 x $rSD_{NEGATIVEPOPULATION}$), where NK-cells were the positive reference population and basophils were the negative reference population.

Abbreviations (alphabetical order): MFI, median fluorescence intensity; NK, natural killer; PE, phycoerythrin; PE-Cy7, phycoerythrin-cyanine 7; rSD, robust standard deviation; SI, stain index.

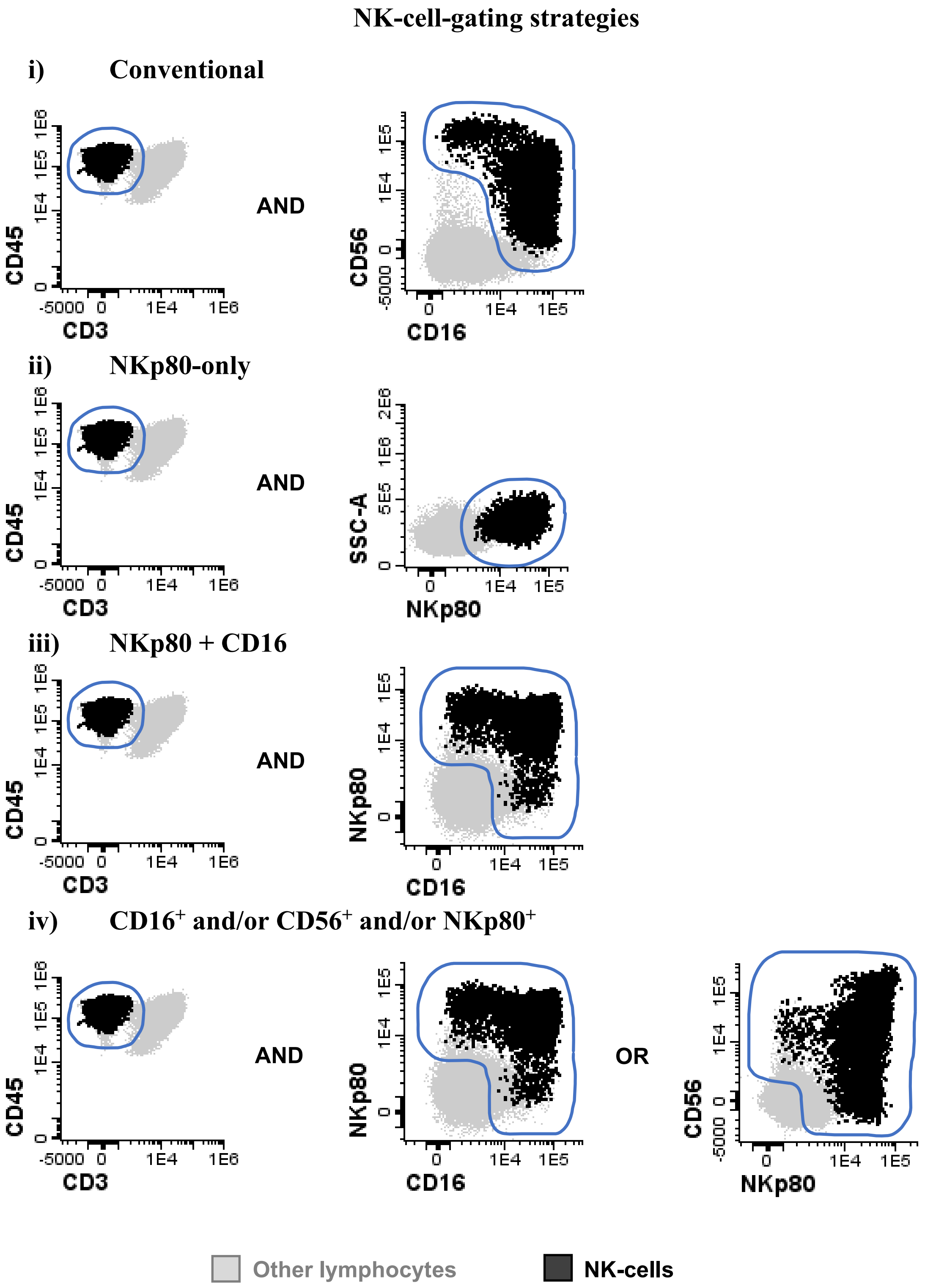

Figure S3 | Different gating approaches used for the identification of NK-cells in human blood. The four strategies applied in parallel in individual blood sample for the identification of NK-cells are shown in a representative sample from a healthy child: i) conventional strategy ($FSC^{lo}SSC^{lo}CD45^{hi}CD16^{-/+}CD56^{lo/+/++}CD3^{-}$); ii) NKp80-only ($FSC^{lo}SSC^{lo}CD45^{hi}NKp80^{+}CD3^{-}$); iii) NKp80+CD16 ($FSC^{lo}SSC^{lo}CD45^{hi}CD16^{-/+}NKp80^{-/+}CD3^{-}$); and iv) all the three markers ($FSC^{lo}SSC^{lo}CD45^{hi}$ $CD3^{-}CD16^{+}$ or $CD56^{+}$ or $NKp80^{+}$).

A

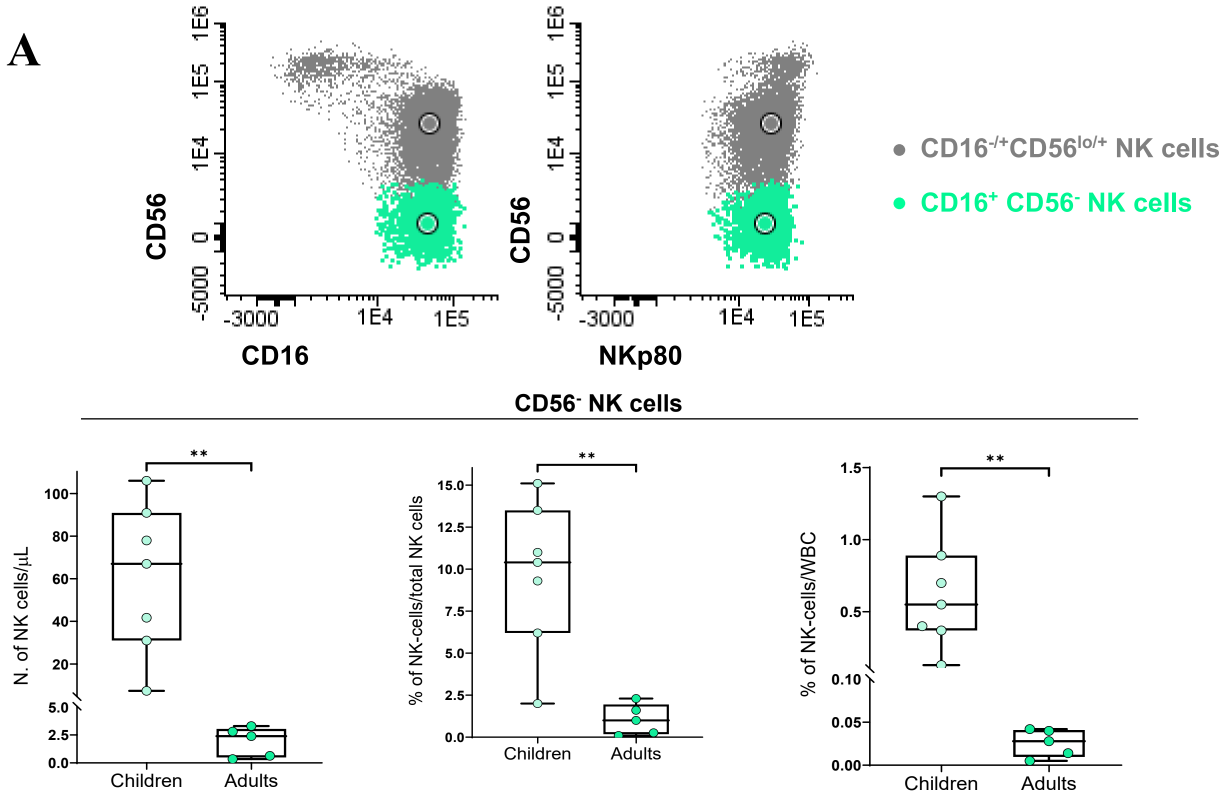

	N. of NK-cells/µL Median (range)	*% from total NK-cells* Median (range)	*% of NK-cells/ WBC* Median (range)
Children *(n=7)*	67 (7.4-106)	10 (2.0-15)	0.55 (0.13-1.3)
Adults *(n=5)*	2.4 (0.35-3.3)	1 (0.1-2.3)	0.03 (0.005-0.04)

B

NKp80^{-} cells with a typical CD16^{+} and/or CD56^{+} NK-cell profile

(Non-T) NKp80^{+} cells outside the typical CD16^{+} and/or CD56^{+} NK-cell profile

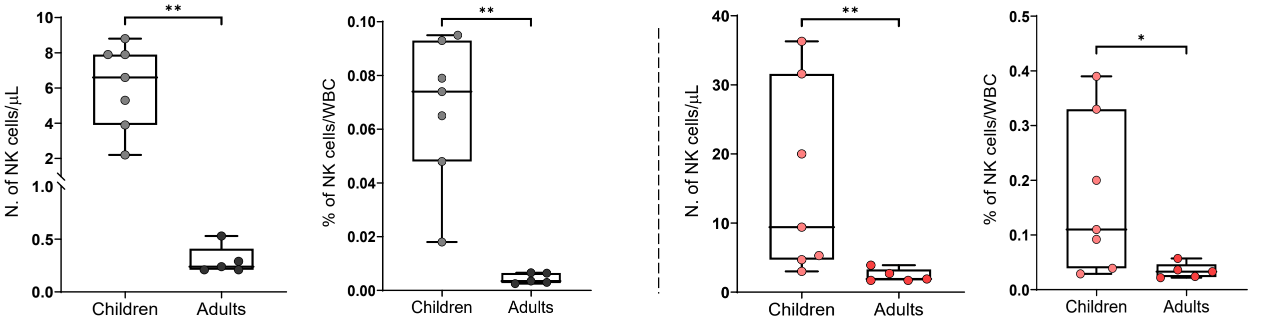

		NK-cells/µL Median (range)	*Percentage of NK-cells/WBC* Median (range)
NKp80^{-} cells with a typical CD16^{+} and/or CD56^{+}profile	**Children** (n=7)	6.6 (2.2-8.8)	0.07 (0.02-0.10)
	**Adults** (n=5)	0.24 (0.21-0.53)	0.004 (0.003-0.00)
Non-T NKp80^{+} cells outside the typical CD16^{+} and/or CD56^{+} pattern	**Children** (n=7)	9.4 (3.0-36)	0.11 (0.03-0.39)
	**Adults** (n=5)	1.9 (1.7-3.9)	0.03 (0.02-0.06)

Figure S4 | NK-cell populations identified with the NKp80 marker in combination with the classical CD56 and CD16 markers used for the identification of blood circulating NK-cells. **(A)** Representative dot plots showing the $CD56^-$ ($CD16^+CD56^-$) NK-cell population (green dots) in blood from a child (upper panels where the larger circles represent the median of each population). Box plots representing the number of $CD16^+CD56^-$ NK-cells/µL, the percentage of $CD16^+CD56^-$ NK-cells among total NK-cells and the percentage of $CD16^+CD56^-$ NK-cells among WBC, both in children and adults are also shown in the lower panels, along with a table displaying the median values (range) obtained for these NK-cell populations. **(B)** Box plots representing the numerical distribution of $NKp80^-$ cells showing the typical $CD16^+$ and/or $CD56^+$ NK-cell profile and the non-T $NKp80^+$ cells outside the classical NK-cell gate of $CD16^+$ and/or $CD56^+$ cells observed in children and adults, expressed as N. of cells/µL and their percentage from WBC. A table with the median values (range) for these minor populations is shown below. In box plots shown in panels **A** and **B**, dots correspond to individual data, while notched boxes represent the 25th and 75th percentile values; lines inside the box correspond to median values (50th percentile) and whiskers represent the minimum and maximum values. *p-value ≤ 0.05; **p-value ≤ 0.01.

Abbreviations (alphabetical order): max, maximum; min, minimum; N., number; neg, negative (lack of expression); NK, natural killer; pos, positive (expression); WBC, white blood cells.

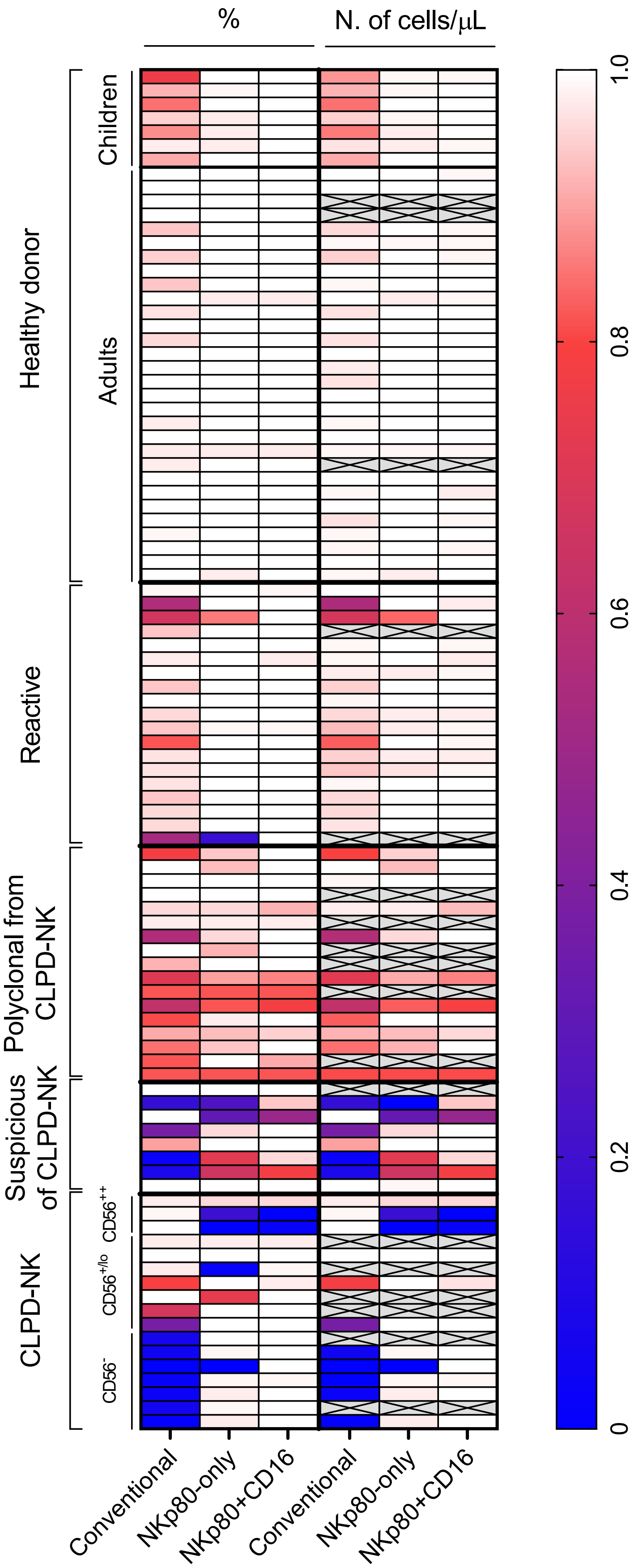
%
N. of cells/μL
Healthy donor
Children
Adults
Reactive
Polyclonal from CLPD-NK
Suspicious of CLPD-NK
CLPD-NK
CD56++
CD56+/lo
CD56-
Conventional
NKp80-only
NKp80+CD16
Conventional
NKp80-only
NKp80+CD16
0
0.2
0.4
0.6
0.8
1.0

Figure S5 | Heatmap representing the number of NK-cells identified in blood of healthy donors and patients with reactive disease condition, suspected CLPD-NK and CLPD-NK, with the different NK-cell gating strategies used vs the reference $CD16^{+}$ and/or $CD56^{+}$ and/or $NKp80^{+}$ approach. Data represented in the heatmap corresponds to the ratio between the number of NK-cells identified either with the conventional strategy ($FSC^{lo}SSC^{lo}CD45^{hi}CD16^{-/+}CD56^{lo/+/++}CD3^{-}$), the NKp80 marker alone and the NKp80 plus CD16 NK-cell gating approach, and the CD56/CD16/NKp80-based reference strategy, based on all three markers used to identify NK-cell populations. The following samples are represented in the heatmap: 37 HD including 7 children and 30 adults, 19 reactive cases, residual polyclonal NK-cells from 17 CLPD-NK cases, as well as NK-cells from 8 cases suspected CLPD-NK and 17 CLPD-NK. CLPD-NK cases are divided into cases carrying clonal $CD56^{++}$ (n=3), $CD56^{-/lo}$ (n=7) and $CD56^{-}$ (n=7) NK-cells. Crossed gray cells in the heatmap represent cases without information about the absolute NK-cell count in blood.

Abbreviations (alphabetical order): CLPD-NK, chronic lymphoproliferative disorder of NK-cells / NK large granular lymphocytic leukemia; HD, healthy donor; lo, low; N., number; NK, natural killer.

CD56^{bright} NK-cells **CD56^{lo} NK-cells**

CD16

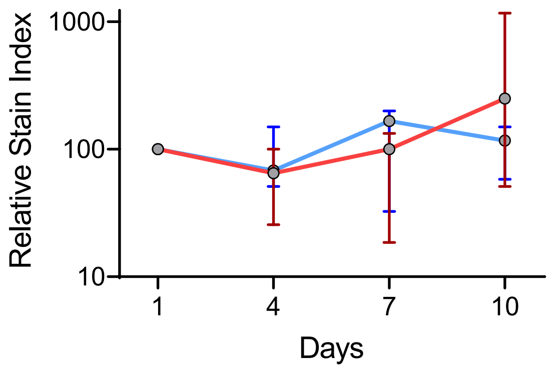

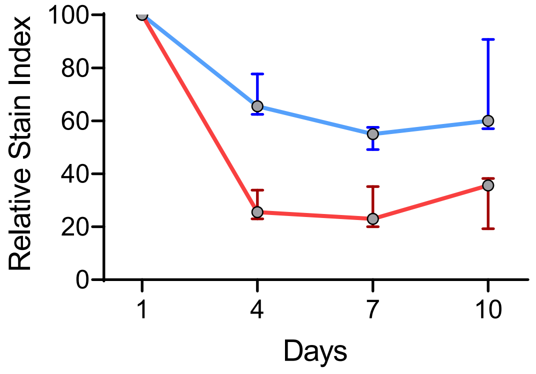

CD56

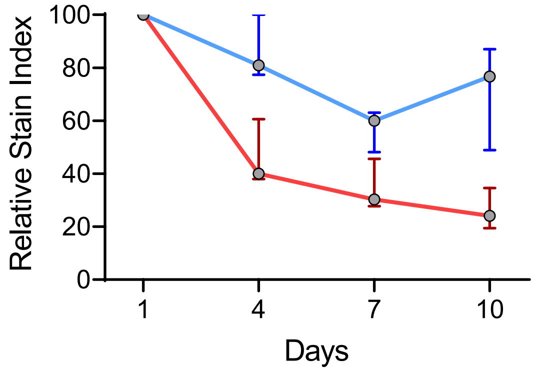

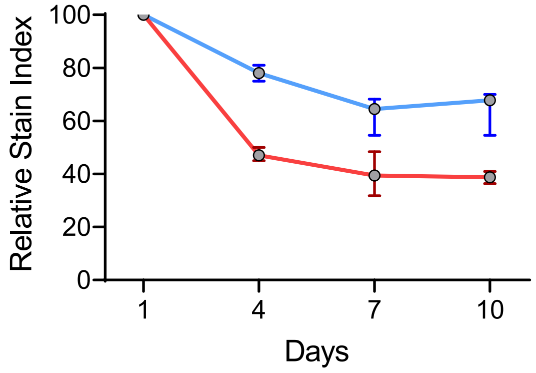

NKp80

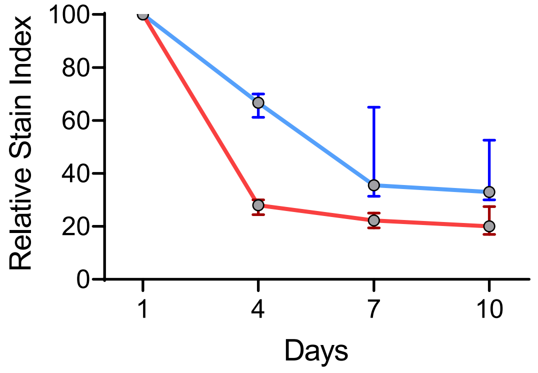

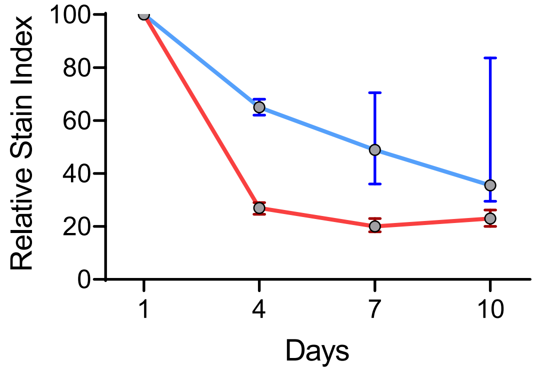

4°C **RT**

Figure S6 | Variation of the stain index of CD16, CD56 and NKp80 on the two major $CD56^{bright}$ and $CD56^{lo}$ NK-cells populations, identified with the conventional strategy ($FSC^{lo}SSC^{lo}CD45^{hi}CD16^{-/+}CD56^{++}CD3^{-}$ or $FSC^{lo}SSC^{lo}CD45^{hi}CD16^{+}CD56^{lo/+}CD3^{-}$, respectively) in blood samples exposed to prolonged periods of storage at different temperatures. Three different samples were stored over time at two different temperatures (4°C and RT) and studied at days +1, +4, +7 and +10, to assess the variation in the SI of CD16, CD56 and NKp80. A viability marker was included to analyse the expression of the NK-cell markers specifically in live cells. Results are shown as median and range (min-max), represented by dots and vertical lines, respectively. Data represent variations (in percentage) of the SI at every time-point, relative to day +1 (where the SI was adjusted to 100%).

Abbreviations (alphabetical order): max, maximum; min, minimum; NK, natural killer; RT, room temperature; SI, stain index.

A

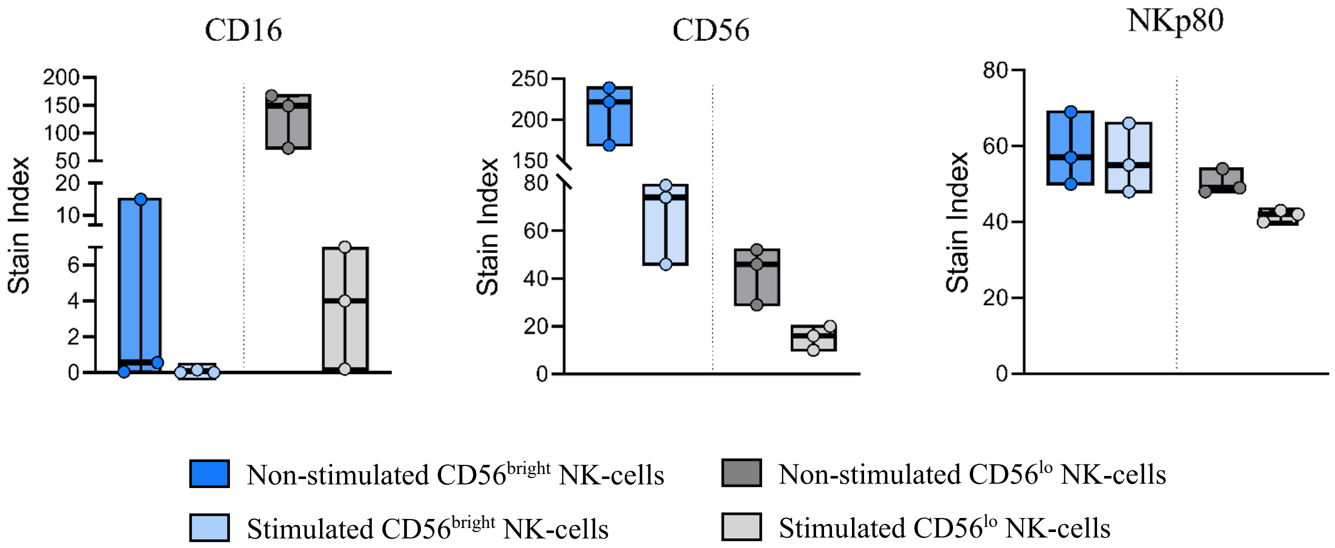
CD16
CD56
NKp80
Stain Index
Non-stimulated CD56bright NK-cells
Stimulated CD56bright NK-cells
Non-stimulated CD56lo NK-cells
Stimulated CD56lo NK-cells

B

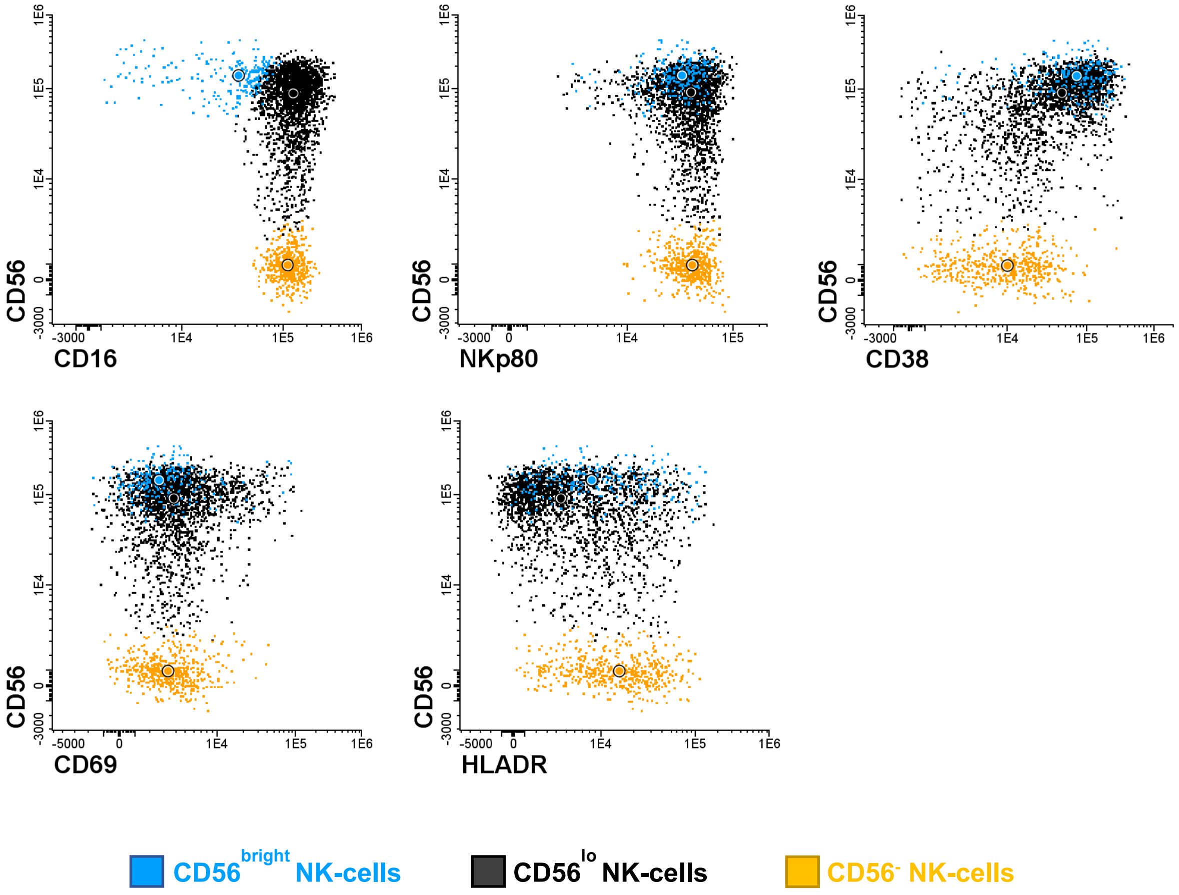
CD56
CD16
NKp80
CD38
CD69
HLADR
CD56bright NK-cells
CD56lo NK-cells
CD56- NK-cells

Figure S7 | Expression pattern of NK-cell markers (CD16, CD56 and NKp80) on activated NK-cells identified with the conventional strategy ($FSC^{lo}SSC^{lo}CD45^{hi}CD16^{-/+}CD56^{lo/+/++}CD3^{-}$). **(A)** Box plots showing the decrease in terms of SI of CD16, CD56 and NKp80 in blood samples after *in vitro* culture. A total of 3 samples were stimulated *in vitro* with PMA and ionomycin, with a parallel unstimulated control. **(B)** Representative dot plots from a peripheral blood sample from a patient with a reactive process (chronic viral infection) showing the performance of CD16, CD56 and NKp80 together with the CD38, CD69 and HLADR activation markers (2,3). Colors represent the different major subsets of NK-cells, blue for $CD56^{bright}$ NK-cells, black for $CD56^{lo}$ NK-cells and yellow for $CD56^{-}$ NK-cells. For each population, the dots represent the median fluorescence channel.

Abbreviations (alphabetical order): NK, natural killer; PMA, phorbol 12-myristate 13 acetate; SI, stain index.

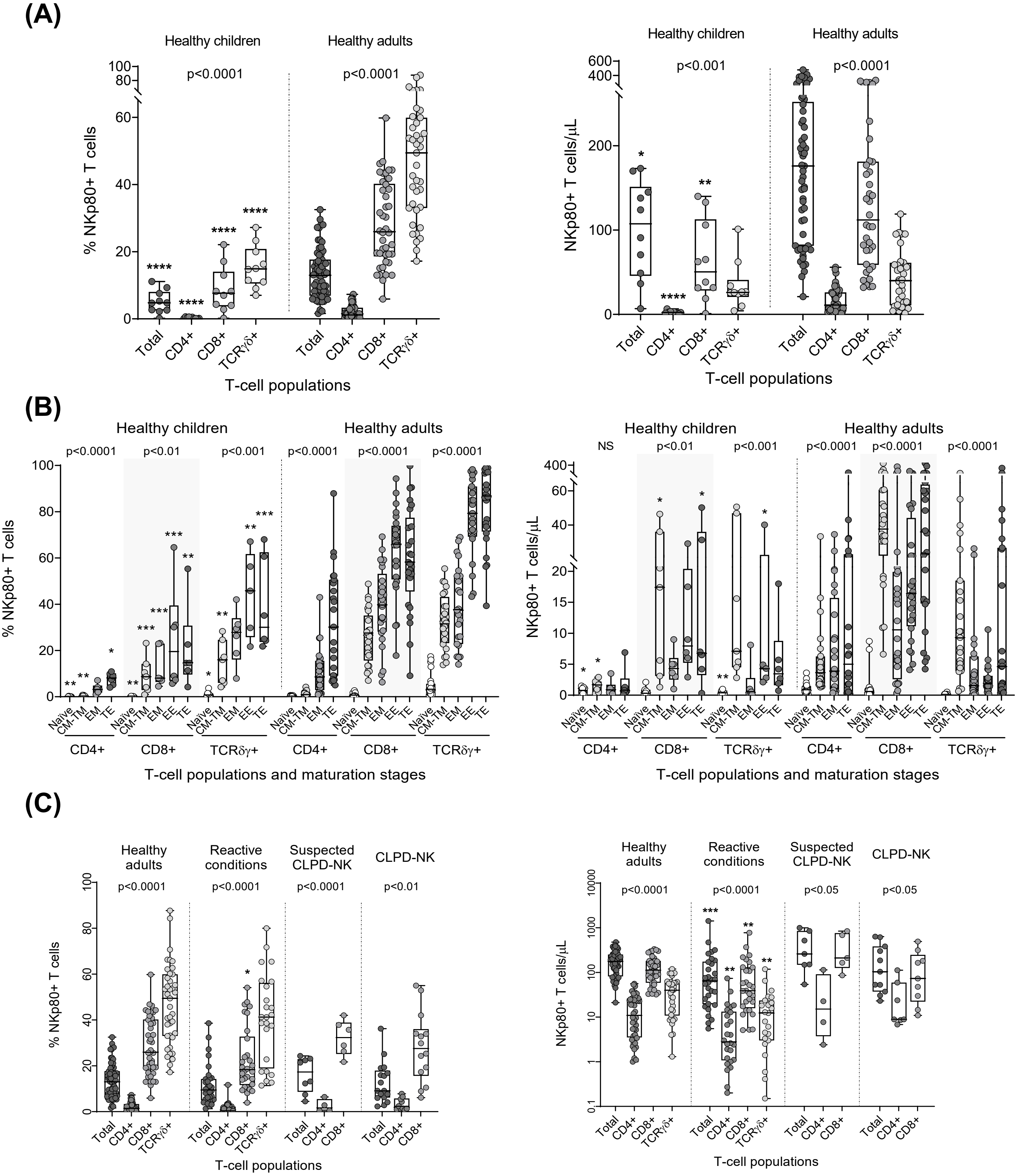
(A)
Healthy children
Healthy adults
p<0.0001
p<0.001
% NKp80+ T cells
NKp80+ T cells/µL
Total
CD4+
CD8+
TCRγδ+
T-cell populations
(B)
Healthy children
Healthy adults
NS
p<0.01
p<0.001
Naïve
CM-TM
EM
EE
TE
CD4+
CD8+
TCRδγ+
T-cell populations and maturation stages
(C)
Healthy adults
Reactive conditions
Suspected CLPD-NK
CLPD-NK
p<0.05
T-cell populations

Figure S8 | Distribution of NKp80$^+$ cells within the different subsets of blood T-cells from HD grouped by age (adults vs children) and the underlying T-cell maturation stages, compared to blood NK-cells from individuals diagnosed with different reactive conditions/diseases or CLPD-NK. **(A)** Box plots show the percentage (from the corresponding T-cell subset) and absolute (cells/µL) numbers of NKp80 positive T-cells among the whole population of T-cells and their TCD4+, TCD8+ and TCRγδ+ T-cell subpopulations present in blood of HD, grouped by age (adults vs children) (*p-value ≤ 0.05, **p-value ≤ 0.01 and ***p-value ≤ 0.001 vs. the corresponding cell subset in adults). **(B)** Box plots show the percentage (from the corresponding T-cell subset) and absolute (cells/µL) number of NKp80$^+$ T-cells in the above-mentioned age groups, grouped by T-cell maturation stages (naïve, CD27$^+$CD45RA$^+$; central/transitional memory, CD27$^+$CD45RA$^-$; effector memory, CD27$^-$CD45RA$^-$; early effector, CD27^{lo}CD45RA$^+$; and terminal effector, CD27$^-$CD45RA$^+$) (*p-value ≤ 0.05, **p-value ≤ 0.01, ***p-value ≤ 0.001 vs. the corresponding cell subset in adults). **(C)** Box plots show the percentage (from the corresponding T-cell subset) and absolute (cells/µL) numbers of NKp80$^+$ T-cells in blood of healthy adults, and individuals with different (reactive) disease conditions (*p-value ≤ 0.05, **p-value ≤ 0.01, ***p-value ≤ 0.001 vs. adult HD). In the box plots, dots correspond to individual data, while notched boxes represent 25th and 75th percentile values; lines inside the box correspond to median values (50th percentiles) and whiskers represent the minimum and maximum values.

Abbreviations (alphabetical order): CM-TM, central memory - transitional memory; CLPD-NK, chronic lymphoproliferative disorder of NK-cells; HD, healthy donor; EE, early effector; EM, effector memory; TE, terminal effector.

REFERENCES

1. Botafogo, V., Pérez-Andres, M., Jara-Acevedo, M., Bárcena, P., Grigore, G., Hernández-Delgado, A., Damasceno, D., Comans, S., Blanco, E., Romero, A., *et al.* (2020). Age Distribution of Multiple Functionally Relevant Subsets of CD4+ T Cells in Human Blood Using a Standardized and Validated 14-Color EuroFlow Immune Monitoring Tube. Front. Immunol. *11*, 495364. 10.3389/FIMMU.2020.00166/BIBTEX.
2. Bárcena, P., Jara-Acevedo, M., Tabernero, M. D., López, A., Sánchez, M. L., García-Montero, A. C., Muñoz-García, N., Vidriales, M. B., Paiva, A., Lecrevisse, Q., Lima, M., Langerak, A. W., Böttcher, S., van Dongen, J. J., Orfao, A., & Almeida, J. (2015). Phenotypic profile of expanded NK cells in chronic lymphoproliferative disorders: a surrogate marker for NK-cell clonality. Oncotarget, 6(40), 42938–42951. doi.org/10.18632/oncotarget.5480
3. Lima, M., Almeida, J., dos Anjos Teixeira, M., Queirós, M. L., Justiça, B., & Orfão, A. (2002). The "ex vivo" patterns of CD2/CD7, CD57/CD11c, CD38/CD11b, CD45RA/CD45RO, and CD11a/HLA-DR expression identify acute/early and chronic/late NK-cell activation states. Blood cells, molecules & diseases, 28(2), 181–190. https://doi.org/10.1006/bcmd.2002.0506
